# Combining hydrophilic chemotherapy and hydrophobic phytotherapy via tumor-targeted albumin–QDs nano-hybrids: covalent coupling and phospholipid complexation approaches

**DOI:** 10.1186/s12951-019-0445-7

**Published:** 2019-01-19

**Authors:** Dina G. Zayed, Shaker M. Ebrahim, Maged W. Helmy, Sherine N. Khattab, Mohammed Bahey-El-Din, Jia-You Fang, Kadria A. Elkhodairy, Ahmed O. Elzoghby

**Affiliations:** 1 Cancer Nanotechnology Research Laboratory (CNRL), Faculty of Pharmacy, Alexandria University, Alexandria, 21521 Egypt; 2 Department of Industrial Pharmacy, Faculty of Pharmacy, Alexandria University, Alexandria, 21521 Egypt; 3 Department of Materials Science, Institute of Graduate Studies and Research, Alexandria University, Alexandria, 21526 Egypt; 4 Department of Pharmacology and Toxicology, Faculty of Pharmacy, Damanhour University, Damanhur, Egypt; 5 Department of Chemistry, Faculty of Science, Alexandria University, Alexandria, 21321 Egypt; 6 Department of Microbiology and Immunology, Faculty of Pharmacy, Alexandria University, Alexandria, 21521 Egypt; 7 Pharmaceutics Laboratory, Graduate Institute of Natural Products, Chang Gung University, Taoyuan, 333 Taiwan; 8 Research Center for Industry of Human Ecology and Research Center for Chinese Herbal Medicine, Chang Gung University of Science and Technology, Kweishan, Taoyuan, 333 Taiwan; 9 Department of Anesthesiology, Chang Gung Memorial Hospital, Kweishan, Taoyuan, 333 Taiwan; 10 Present Address: Division of Engineering in Medicine, Department of Medicine, Brigham and Women’s Hospital, Harvard Medical School, Boston, MA 02115 USA; 11 Harvard-MIT Division of Health Sciences and Technology, Cambridge, MA 02139 USA

**Keywords:** Albumin nanoparticles, QDs, Resveratrol, Pemetrexed, Mannose targeting, Breast cancer, Theranostics

## Abstract

**Background:**

The rationale of this study is to combine the merits of both albumin nanoparticles and quantum dots (QDs) in improved drug tumor accumulation and strong fluorescence imaging capability into one carrier. However, premature drug release from protein nanoparticles and high toxicity of QDs due to heavy metal leakage are among challenging hurdles. Following this platform, we developed cancer nano-theranostics by coupling biocompatible albumin backbone to CdTe QDs and mannose moieties to enhance tumor targeting and reduce QDs toxicity. The chemotherapeutic water soluble drug pemetrexed (PMT) was conjugated via tumor-cleavable bond to the albumin backbone for tumor site-specific release. In combination, the herbal hydrophobic drug resveratrol (RSV) was preformulated as phospholipid complex which enabled its physical encapsulation into albumin nanoparticles.

**Results:**

Albumin–QDs theranostics showed enhanced cytotoxicity and internalization into breast cancer cells that could be traced by virtue of their high fluorescence quantum yield and excellent imaging capacity. In vivo, the nanocarriers demonstrated superior anti-tumor effects including reduced tumor volume, increased apoptosis, and inhibited angiogenesis in addition to non-immunogenic response. Moreover, in vivo bioimaging test demonstrated excellent tumor-specific accumulation of targeted nanocarriers via QDs-mediated fluorescence.

**Conclusion:**

Mannose-grafted strategy and QD-fluorescence capability were beneficial to deliver albumin nanocarriers to tumor tissues and then to release the anticancer drugs for killing cancer cells as well as enabling tumor imaging facility. Overall, we believe albumin–QDs nanoplatform could be a potential nano-theranostic for bioimaging and targeted breast cancer therapy.

**Electronic supplementary material:**

The online version of this article (10.1186/s12951-019-0445-7) contains supplementary material, which is available to authorized users.

## Background

Nano-theranostics can provide both therapeutic and diagnostic capabilities via a singular drug nanocarrier [1]. Limitations of current imaging techniques lead to design of sensitive and biospecific new imaging probes. Quantum dots (QDs) as new imaging probes, 2–10 nm in diameter. They possess strong photoluminescence (PL) with a high molar extinction coefficient values compared with organic dyes in addition to broad absorption with narrow symmetric emission spectra. Some concerns were raised regarding QDs toxicity, in particular for cadmium-containing QDs due to the release of Cd ions and reactive oxygen species (ROS) generation [2]. Other studies confirmed that ROS generated by QDs help induce more apoptosis and thus converted the QDs toxicity into a therapeutic modality [3]. Recently, great interest has been raised towards the fabrication of cadmium-free QDs to be used for biological applications such as ZnS and ZnO QDs; unfortunately, they exhibited weaker PL emission than Cd-containing QDs. Therefore, researchers are now paying more effort to decreasing toxicity of the highly fluorescent Cd-containing QDs.

Among the most outstanding strategies to reduce their toxicity, QDs were conjugated via tumor-cleavable bond to actively-targeted delivery systems to maximize their accumulation in tumor cells and inhibit their release into circulation [4]. Similar literature reported the tumor-specific release of QDs coupled to NPs by amide bond. Methotrexate (MTX) was covalently conjugated to l-cysteine capped CdSe QDs through a strong amide bond forming MTX-QD nano-conjugates, this conjugate can’t be cleaved in PBS. However, once taken up by the cells, the amide bond is enzymatically cleaved by the action of the endosomal/lysosomal machineries of the target cells and thus releasing MTX molecules and the QDs into the cytoplasmic milieu of the cells. Cellular uptake study revealed that 71% of MTX-QD nanoconjugate accumulated in KB cancer cells [5]. Receptor-targeted nano-sized carriers can facilitate tumor targeting via the enhanced tumor permeability and retention (EPR) effect, followed by binding to receptors over-expressed by cancer cells leading to enhanced internalization via endocytosis [6, 7]. Cancer cells were found to overexpress C-type lectin receptors (CLRs) essentially required for binding with carbohydrates such as mannose. It is widely known that mannose bind strongly to mannose receptor (MR) overexpressed on the surface of human breast cancerous cells [8]. However, the interaction between a single mannose and MR is weak thus nanoparticles can provide a platform for the multivalent presentation of mannose, which increases the binding intensity and specificity between mannose-functionalized nanocarriers and overexpressed MRs on cancer cells [9].

Pemetrexed (PMT) is an antifolate cytotoxic drug delivered into cells via folate transport systems where it is transformed to polyglutamate derivatives by folyl-polyglutamate synthase [10]. Unlike methotrexate which targets a single enzyme critical in purine and pyrimidine synthesis, PMT and its polyglutamate derivatives inhibit three enzymes; dihydrofolate reductase, thymidylates synthase, and glycinamide ribonucleotide formyltransferase. The multiple enzyme inhibitory effect of PMT helps overcoming intrinsic resistance accompanied by mutation of one of these enzymes. However, the relative lack of PMT specificity resulted in toxic dose-related adverse effects that may limit its therapeutic effect such as myelosuppression, hepatic enzyme elevations, maculopapular rash, emesis and diarrhea. To reduce its toxicity, PMT was encapsulated into nanocarriers including PEG–peptide–PCL NPs [11] and lipid–polymer hybrid NPs [12]. The small size (below 100 nm)of NPs containing both PMT and miR-21 antisense oligonucleotide, enabled their passive tumor targeting and enhanced uptake into U87MG human glioblastoma cells [12]. On the other hand, Resveratrol (RSV), a natural polyphenol and phytoalexin, demonstrated a strong anti-cancer activity. However, its low aqueous solubility and poor bioavailability hinder its clinical utility. To overcome this challenge, RSV was incorporated into different nano-formulations to enhance its solubility and bioavailability [13].

Different from cancer monotherapy, combination therapy has the advantage of harmonizing different signaling pathways as well as amplifying the therapeutic effect by overcoming resistance to cancer monotherapy [14]. In our laboratory, co-loading of celecoxib and letrozole into protamine-coated oily-core nanocapsules enhanced their anti-cancer efficacy against breast cancer cells in vitro and in vivo compared to single free drugs [15]. The combined treatment with RSV and PMT was previously reported to demonstrate a synergistic growth inhibition of human NSCLC H520 and H1975 cells compared to either treatment alone [16]. On another avenue, co-treatment of MSTO-211 and A549 cells with RSV and PMT was found to reduce ROS generation in both cells more efficiently than in cells treated with PMT alone. Therefore, in addition to synergism, it was postulated that RSV may confer protection against apoptosis induced by PMT so help decrease PMT toxicity [17].

In this study, we propose for the first time up to our knowledge, mannosylated BSA-QDs nano-theranostics for targeted co-delivery of PMT and RSV to breast cancer. First, the hydrophilic cytotoxic drug, PMT, was covalently-bonded to the surface of BSA via amide bond stable at systemic circulation but can be cleaved at tumor cells thus enabling its specific release at tumor sites and reducing its systemic toxicity. Second, to overcome its high lipophilicity, RSV was pre-formulated as phosphatidylcholine complex (RSV-PC) prior to incorporation into the hydrophilic matrix of BSA NPs. Third, to achieve active tumor-targeting, the surface of BSA NPs was decorated with d-mannose for binding to MRs overexpressed by human breast cancer cells. Finally, for theranostic applications, water-soluble thiol capped CdTe QDs were conjugated to the surface of BSA NPs via tumor cleavable bond to inhibit release of Cd ions into circulation thus reducing QDs toxicity. The developed delivery system was thoroughly investigated in vitro and in vivo to prove the anti-tumor superiority of the combined drug nanocarriers compared with free drugs and their combination.

## Materials and methods

### Materials

Resveratrol (RSV) and pemetrexed-disodium (PMT) were purchased from Xi’an natural field bio-technique Co., Ltd. (Shaanxi, China). Bovine serum albumin (BSA), sodium borohydride (NaBH_4_), cadmium chloride (CdCl_2_·2.5H_2_O), sodium hydroxide, disodium hydrogen phosphate (Na_2_HPO_4_), thioglycolic acid (TGA), tellurium powder (99.999%), fetal bovine serum (FBS), 3-(4,5-dimethylthiazolyl-2)-2,5-diphenyltetrazolium bromide (MTT), mannitol, d-mannose, dimethyl sulfoxide (DMSO), *N*-(3-dimethylaminopropyl), *N*′-ethylcarbodiimide hydrochloride (EDC·HCl) (purity > 98%), *N*-hydroxysuccinimide (NHS), Triton X100, haematoxylin solution, eosin solution and Canada balsam were purchased from Sigma–Aldrich (St. Louis, USA). 2-(4-ethoxyphenyl)-6-[6-(4-methylpiperazin-1-yl)-1*H*-benzimidazol-2-yl]-1*H*-benzimidazole (Hoechst) was purchased from Thermo-Fisher (Waltham, MA, USA). DPX mounting medium was obtained from Loba Chemie Pvt. Ltd. (Mumbai, India). Fat-free soybean phospholipids with 70% phosphatidylcholine (Lipoid S75) were kindly provided by Lipoid (Ludwigshafen, Germany). Absolute ethanol, methanol, tertiary butyl alcohol (TBA) and orthophosphoric acid were from ADWIC, El-Nasr Pharmaceutical Chemicals Co., (Cairo, Egypt). Acetonitrile HPLC grade was obtained from JT Baker (Phillipsburg, NJ, USA). Peroxidase-conjugated anti-mouse IgG and 3,3′,5,5′-tetramethylbenzidine (TMB) were purchased from KPLInc., (Gaithersburg, USA). Mouse anti-Ki-67 monoclonal antibody was obtained from Santa Cruz Biotechnology (Cat no. sc-23900). Human MCF-7 and MDA-MB-231breast adenocarcinoma cells were supplied by the American Type Culture Collection (ATCC).

### Synthesis of CdTe QDs

Water soluble TGA-capped CdTe QDs were synthesized in accordance with the modified coordinating solvent method [18]. Briefly, 0.158 g CdCl_2_·2.5H_2_O and 150 µl TGA were dissolved in 100 ml deionized water. The solution pH was adjusted to 10.0 using 1.0 M NaOH solution. This Cd source solution was then deoxygenated in a three-neck flask by bubbling nitrogen gas for at least 30 min and refluxed at 100 °C. Meanwhile, 0.06 g Te powder and 0.07 g NaBH_4_ were mixed into 10 ml deionized water forming a black mixture. The mixture was then continuously stirred under nitrogen gas at 60 °C until the black color disappeared and the purple colored NaHTe was obtained. The recently prepared NaHTe solution was then injected into the three-neck flask under nitrogen atmosphere. CdTe QDs start to form and grow upon refluxing at 100 °C under nitrogen atmosphere for 2 h with a condenser attached. Samples of the reaction mixture solution were taken at specified time intervals (5:120 min) and the absorption spectrum was taken to monitor the growth of the clusters.

### Characterization of CdTe QDs

The absorption spectra of CdTe QDs were recorded via T80 UV-visible spectrophotometer (PG Instruments, UK). The diameter of synthesized QDs was determined based on the following equation [18]:

1 $$\begin{aligned}D & = 9.8127 \times 10^{ - 7} \lambda^{3} - 1.7147 \times 10^{ - 3} \lambda^{2 } \\ & \quad + 1.0064 \uplambda - 194.84\end{aligned}$$

where D (nm) is the diameter of QDs, and λ (nm) is the wavelength of the first excitonic absorption peak.

The fluorescence intensity and emission spectra were recorded at excitation wavelength of 450 nm by LS 55 fluorescence spectrophotometer (PerkinElmer, USA). TEM analysis as well as the zeta potential of QDs were estimated following the experimental method detailed in Additional file 1.

### Preparation of RSV/PMT-BSA NPs (F1)

PMT–BSA conjugate was prepared by carbodiimide coupling [19] while RSV-PC complex was prepared via freeze-drying technique [20] (Methodology is detailed in Additional file 1). BSA NPs were fabricated by an established desolvation process. Briefly, 100 mg of PMT-BSA conjugate was dissolved in 4.0 ml NaCl solution (10 mM) and adjusted to pH 7.4. RSV-PC complex (eq. to 10 mg RSV) was pre-dissolved in the aqueous BSA-PMT solution and left to incubate under magnetic stirring for 1 h before desolvation step. The NPs were formed by continuous dropwise addition of 8.0 ml ethanol (1 ml/min) under magnetic stirring. 55 µl of 8% v/v glutaraldehyde was then added for crosslinking of the desolvated particles. The formed NPs were left overnight under magnetic stirring and then centrifuged twice at 16,000 rpm for 30 min at 4 °C for purification (3–30 KS Sigma, Germany).

### Preparation of RSV/PMT-BSA-QDs NPs (F2)

For activation of carboxylate groups of TGA-capped QDs, EDC·HCl (0.024 g, 0.12 mmol) and NHS (0.015 g, 0.12 mmol) were added to 2 ml of CdTe QDs dispersion in water (adjusted to pH 7.4 using 0.1 M HCl) under stirring for 30 min. The previously prepared RSV/PMT-BSA NPs solution was added to the reaction solution and left for 24 h under stirring. Finally, the NPs were centrifuged at 16,000 rpm for 30 min at 4 °C to separate the excess un-reacted reagents and redispersed in water to be used for further characterization.

### Preparation of mannose-targeted RSV/PMT-BSA-QDs NPs (F3)

For preparation of mannosylated-BSA NPs, the prepared RSV/PMT-BSA-QDs NPs solution was mixed with 15 mg d-mannose pre-dissolved in 4.0 ml water. The solution pH was adjusted to 4.0 using 0.1 M acetic acid and left in a type 3047 Kottermann shaking water bath (Hanigsen, Germany) at 45 °C for 4 h. Mannosylated BSA NPs solution was then purified by dialysis against deionized water for 24 h, with water being replaced every 3–5 h to remove un-reacted mannose.

### Solid state characterization

The FTIR spectra and DSC thermograms of free drugs and drug-loaded nanocarriers were recorded with the procedures mentioned in Additional file 1 [21, 22]. Moreover, nuclear magnetic resonance (NMR) spectra (^1^H-NMR) were recorded using JEOL 500 MHz spectrometer (Tokyo, Japan) at ambient temperature for BSA, mannose and mannose-BSA NPs. Chemical shifts are recorded in ppm and referenced relative to residual solvent.

### Physicochemical characterization of dual drug-loaded BSANPs

The methodologies for assessing drug loading, nanoparticle size and zeta potential [23], drug release [24], morphology, stability [25], redispersibility, hemolytic and serum stability [26] were performed as described and detailed in Additional file 1.

### In vitro cytotoxicity and uptake study

The in vitro cytotoxicity of free RSV, free PMT, free RSV/PMT solution and different dual drug loaded BSA-QDs NPs against human breast cancer MCF-7 and MDA-MB-231 cells were evaluated by MTT assay performed as described and detailed in Additional file 1. Combination Index (CI) and Dose Reduction Index (DRI) were calculated using CompuSyn software (version 1) to ensure the superiority of different nanocarriers compared to the free combination [6]. Cellular uptake of targeted and non-targeted BSA-QDs NPs and free QDs into MCF-7 breast cancer cells was evaluated using confocal microscopy as described previously [27] and detailed in Additional file 1.

### In vivo studies

#### Animals

The anti-tumor efficacy of drug loaded NPs (F2, F3 and F4) was evaluated compared to free RSV, free PMT and RSV/PMT solution on female mice housed in stainless steel mesh cages following standard protocol mentioned in Additional file 1.

#### Development of tumor model

7–8 weeks aged female BALB/C mice were housed in a pathogen-free environment at 7 mice/cage. They were provided with autoclaved non-fluorescented mouse chow and water. Ehrlich ascites tumor (EAT) cells, given from the National Institute of Cancer, Egypt, were collected from the ascitic fluid of BALB/C mice harboring 8–10 days old ascitic tumor. Almostly, 10^7^ of EAT cells suspended in PBS were injected into the left side of the mammary fat pad of BALB/C female mice. Tumor growth was estimated daily until its volume reached 100 mm^3^. Tumor volume was determined by measuring both perpendicular diameters of the tumor using a micrometer based on the following equation [28]:

2 $$Tumor\;\;volume = L \times W^{2} \times 0.5$$

where W is tumor width, L is tumor length.

#### In vivo anti-tumor efficacy

Animal groups (7 mice each) included untreated positive control, negative control, free RSV, free PMT, free combined RSV/PMT solution, F2 (RSV/PMT-BSA-QDs NPs), F3 (Mann-targeted RSV/PMT BSA-QDs NPs)and F4 (Mann-targeted RSV/PMT BSA NPs) treated group; in addition to blank BSA NPs. Free drugs or NPs equivalent to 4 mg/kg PMT and 2.3 mg/kg RSV were injected i.v. through the tail vein into EAT-bearing mice three times per week for 3 weeks. The mice body weight was recorded simultaneously during the treatment. Animals were sacrificed at the end of treatment period. Tumors were isolated and the weights were determined. Each excised tumor was divided into 2 parts for histopathological examination and measurement of tumor growth biomarkers.

- Tumor volume
  During the treatment course, tumor growth was assessed once per week and the % increase of tumor volume was determined.
- Tumor growth biomarkers
  The tumor growth biomarkers were determined quantitatively using ELISA. The experimental method is detailed in Additional file 1.
- Histopathological and immunohistochemical analysis
  The tumor samples were examined for histopathological changes as well as proliferation extent. The experimental method is detailed in Additional file 1.
- Tumor localization of NPs
  The distribution of BSA-QDs NPs was analyzed using the confocal laser scanning microscopy as described and mentioned in Additional file 1.

#### Immunogenicity of the nano-delivery system

Enzyme-linked immunosorbent assay (ELISA) was used as previously described [29] to check if the BSA nano-delivery system elicits antibodies against BSA during the experimental treatment protocol (Experimental details are described in Additional file 1).

#### Statistical analysis

Data analysis is detailed in Additional file 1.

## Results and discussion

**Fig. 1:**
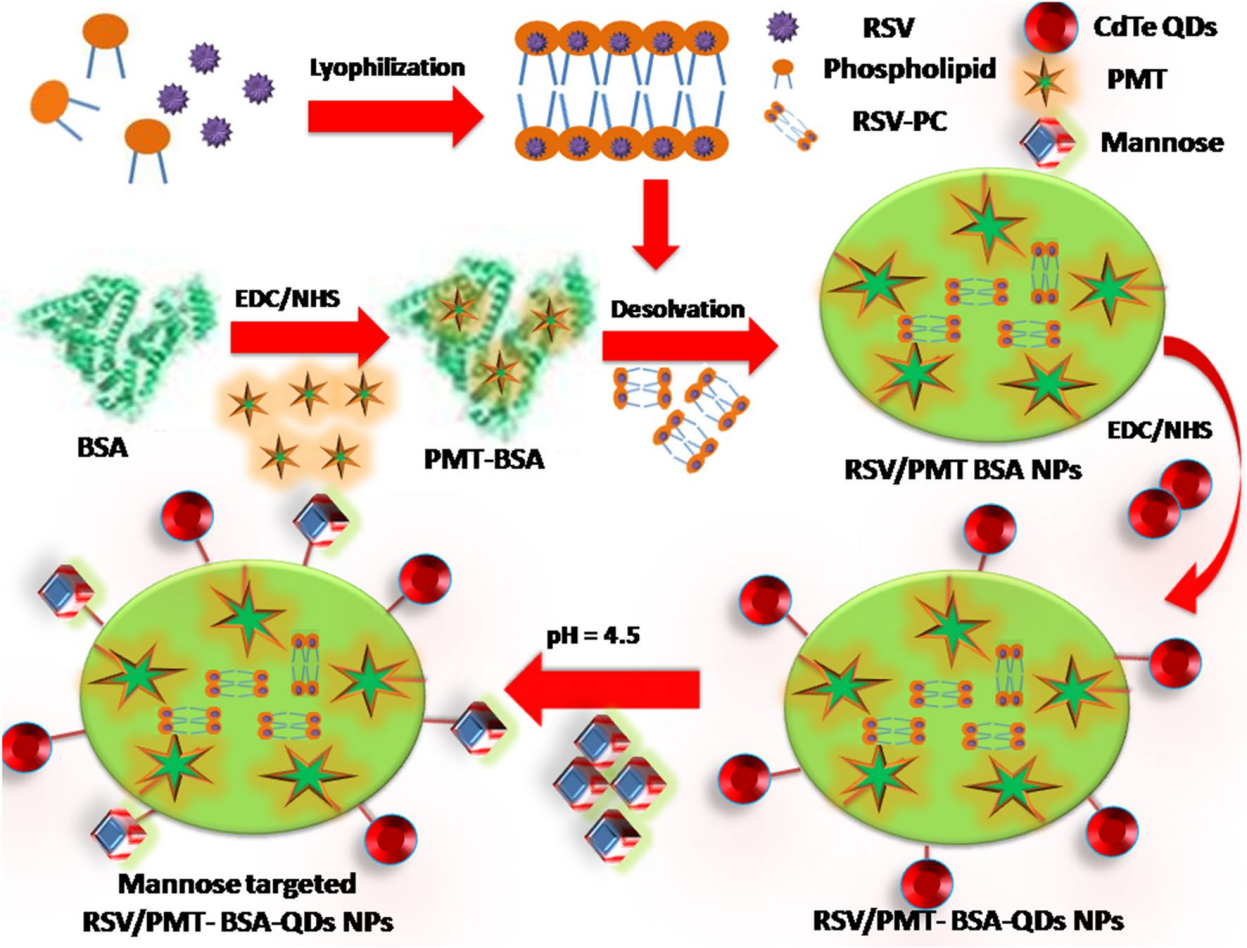
Schematic diagram illustrating the preparation steps of Mann-targeted RSV/PMT BSA-QDs NPs (F3)

Nanocarriers fabricated from natural polymers including proteins and polysaccharides offer various opportunities for tumor-targeted drug delivery applications [30–32]. Albumin can be used as a drug carrier either by incorporation of the drug within its matrix or by conjugation to the functional groups available on the NPs surface. In this study, BSA-QDs NPs for dual delivery of RSV and PMT were developed for breast cancer therapy and imaging (Fig. 1).

### Characterization of the synthesized CdTe QDs

CdTe QDs are characterized by their strong fluorescence enabling their use in cancer imaging. In our study, highly fluorescent QDs have been prepared using 4:1 Cd:Te molar ratio at pH 10.0 utilizing 150 µl TGA as a capping agent. The fluorescence intensity of CdTe QDs was previously reported to increase with increasing Te concentration till reaching maxima at Cd:Te molar ratio of 4:1 [33]. Finally, the solution pH was adjusted to 10.0 as an optimal value. Sun et al. found that the highest fluorescence intensity of QDs was obtained at pH of 10.5 [34].

**Fig. 2:**
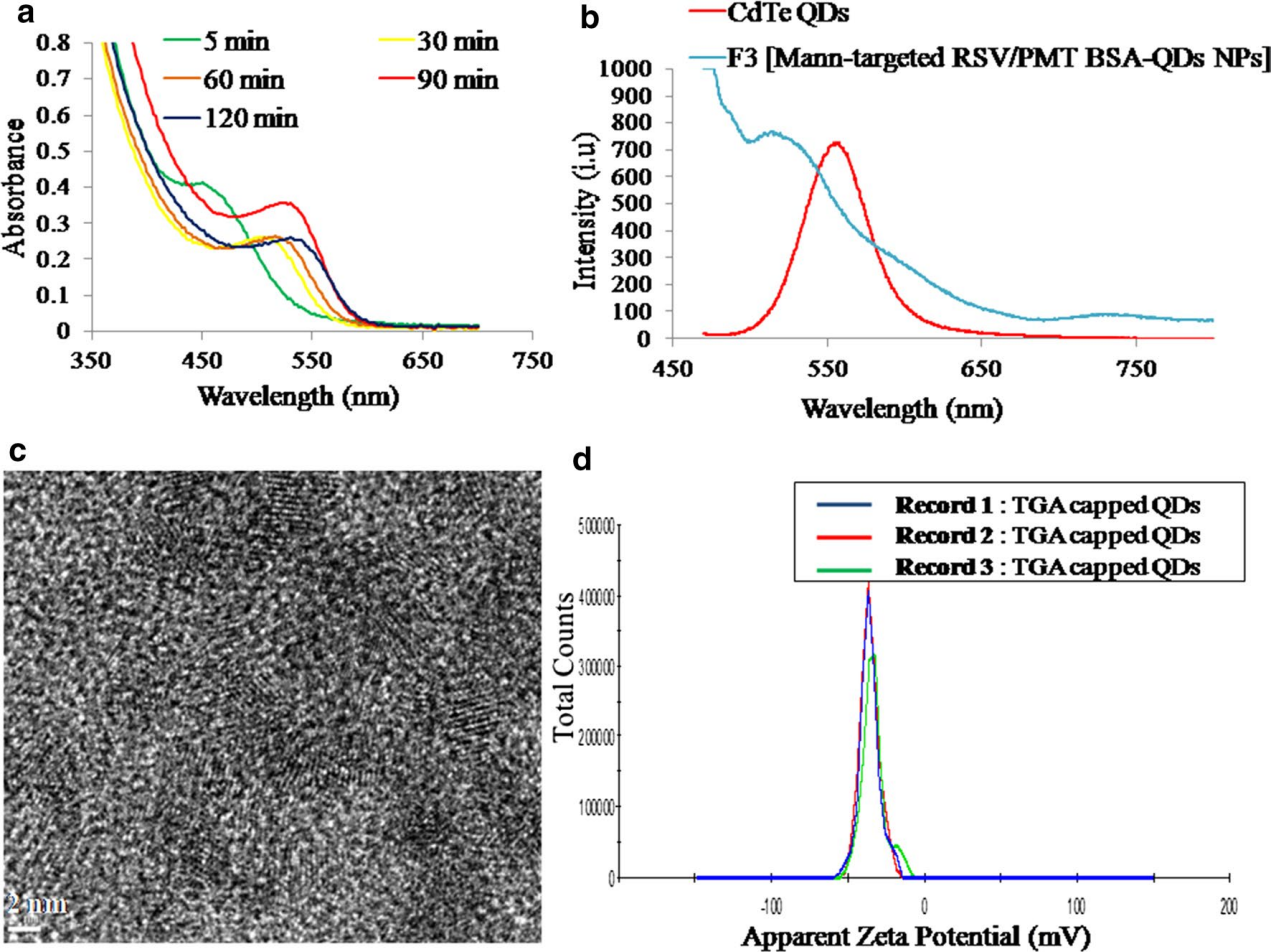
UV absorbance measured during the preparation of TGA capped CdTe QDs (**a**), emission spectra of CdTe QDs and Mann-targeted RSV/PMT BSA-QDs NPs (F3) upon excitation with λ = 450 nm (**b**), TEM images of TGA capped CdTe QDs **(c**) and their corresponding zeta potential distribution diagram (**d**)

During the formation of CdTe QDs, the solution color was changed from colorless to transparent yellow to light brown and finally red brown. This revealed the formation of QDs with different sizes upon increasing the reaction time. From UV–visible spectra of the TGA-capped QDs, it has been observed that QDs started growth at a wavelength of 460 nm (Fig. 2a). As the reaction time passes, the spectra were shifted towards the red region due to the quantum confinement effect indicating the size growth of QDs [18]. From equation one, the size of QDs was calculated to be 3.2 nm at wavelength of 550 nm. At excitation wavelength of 450 nm, the TGA-capped QDs and Mann-targeted RSV/PMT-BSA-QDs NPs (F3) exhibited a characteristic symmetric emission peak at 560 nm (Fig. 2b). The QDs exhibited high adequate emission spectrum along with high resolution that can be utilized for cancer cell imaging [35]. A slight blue shift in the emission peak of CdTe QDs to 520 nm was observed after conjugation with Mann-targeted RSV/PMT-BSA-QDs NPs (F3) (Fig. 2b). During preparation of CdTe QDs, the thiol groups of TGA stabilizer were conjugated to the QDs surface by SH-Cd coordination. Thus, the free carboxylic group of TGA could be easily coupled to the amino groups of BSA resulting in shift of emission peak towards shorter wavelength. This shift confirmed that the size of the QDs was reduced due to etching of nanocrystals by photo-oxidations or acids as a result of decreasing pH. In our study, pure CdTe QDs were prepared at pH 10.0 while during preparation of F3 nanoparticles [Mann-targeted RSV/PMT BSA-QDs NPs], the pH was decreased to 7.0. Similarly, phospholipid micelles encapsulated DHLA QDs showed a spectral blue shift of 5 nm upon decreasing the pH to 5.0 [36]. As illustrated in HRTEM images, TGA-capped CdTe QDs were well dispersed with an average size of 4–5 nm (Fig. 2c). The good dispersion of QDs was also indicated by the high value of their zeta potential (− 35.8 mV) which could be attributed to the free carboxylic groups of TGA (Fig. 2d). The size obtained by HRTEM was similar or slightly higher than that calculated from absorption measurements at wavelength of 550 nm (3.2 nm). TEM measures the diameter of the particle with its surface attached ligands and strongly associated solvent molecules while the size calculated from absorption spectrum is only based on QDs distribution [18].

### Physicochemical characterization of drug-loaded BSA NPs

In our study, to develop dual drug-loaded BSA NPs, the chemotherapeutic water soluble drug, PMT, was firstly conjugated to BSA via carbodiimide coupling reaction. PMT carboxylate groups were activated and reacted with the free amino groups of BSA to form an amide bond. BSA-PMT NPs were prepared by desolvation where addition of ethanol to aqueous BSA-PMT solution diminished its water solubility leading to its precipitation followed by condensation with glutaraldehyde for hardening of the formed coacervates [30]. By virtue of the abundant binding sites of albumin for hydrophobic drugs, we have incorporated the water insoluble herbal drug, RSV, into the hydrophilic albumin matrix [30]. To facilitate its dispersion in aqueous solution, RSV-phospholipid complex (RSV-PC) was formed to enhance the drug water solubility and hence RSV could be incorporated in the albumin matrix. The RSV-PC complex was formed via van der Waals forces and hydrogen bonds between the phospholipid polar head and the polar functionalities of RSV (details of the physicochemical characteristics of RSV-PC complex are in Additional file 1) [22]. Similarly, phospholipid was used for complexation with the water insoluble drug, teniposide to improve its solubility and thus facilitated its incorporation into albumin NPs with encapsulation efficiency of 82.27% [37]. The prepared RSV/PMT-BSA NPs (F1) showed an average size of 142.8 ± 5.72 nm and zeta potential of − 31.7 ± 1.5 mV providing highly electrostatic repulsive stabilization of the particles (Table 1). The encapsulation efficiency of RSV was 65.33 ± 5.8% while the conjugation efficiency of PMT was 63.3 ± 2.1%.

**Table 1:** Composition and physicochemical characteristics of blank and drug-loaded BSA NPs

	Formula	Particle size (nm)	Zeta potential (mV)	PDI	%RSV EE	%PMT CE
F1*	RSV/PMT-BSA NPs	142.8 ± 5.7	− 31.7 ± 1.5	0.158	65.33 ± 5.8	63.3 ± 2.1
F2	RSV/PMT-BSA-QDs NPs	154.8 ± 6.6	− 27.2 ± 1.1	0.202	67.34 ± 6.1	64.2 ± 2.3
F3	Mann-targeted RSV/PMT-BSA-QDs NPs	193.9 ± 4.8	− 33.1 ± 1.2	0.184	68.1 ± 5.1	50.9 ± 1.9
F4	Mann-targeted RSV/PMT-BSA NPs	155.5 ± 3.9	− 32.7 ± 1.4	0.117	65.2 ± 4.9	57.8 ± 3.6

* All NPs were prepared using 100 mg PMT-BSA, RSV/PC complex eq. to 10 mg RSV, and 55 µl (8%v/v) glutaraldehyde as a cross-linking reagent

**Fig. 3:**
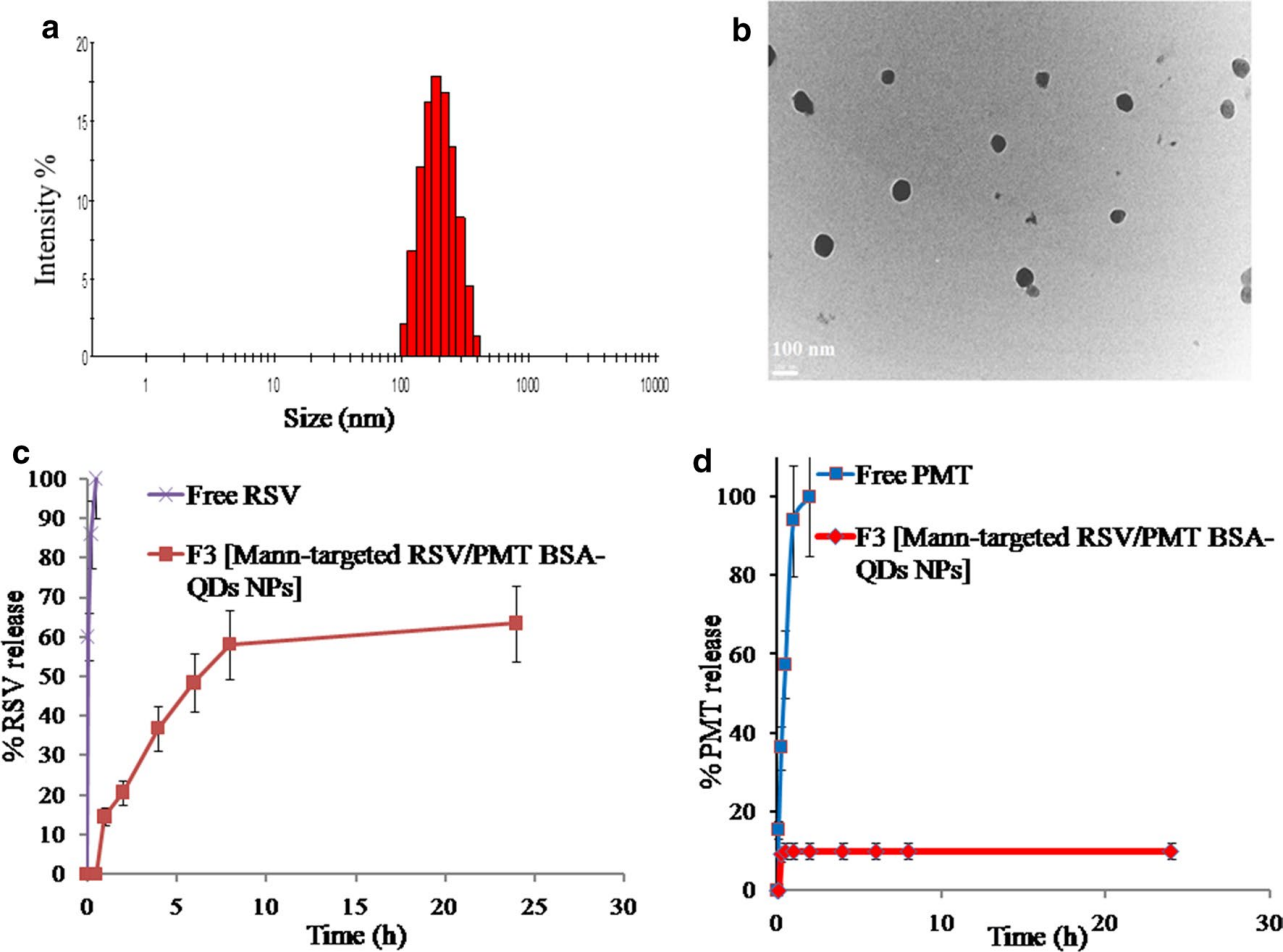
Size distribution diagram of Mann-targeted RSV/PMT BSA-QDs NPs (F3) (**a**), TEM images showing morphology of Mann-targeted RSV/PMT BSA-QDs NPs (F3) (**b**) and in-vitro release study of RSV (**c**) and PMT (**d**) from (F3) in PBS (pH 7.4) at 100 rpm and 37 °C using dialysis bag method

In a consequent step, for development of theranostic NPs, the carboxylate groups of TGA-CdTe QDs were coupled to the surface amino groups of dual drug-loaded BSA NPs to elaborate albumin-inorganic nano-hybrids. RSV/PMT-BSA-QDs NPs (F2) demonstrated a size of 154.8 ± 6.6 nm with a surface charge of -27 ± 1.1 mV (Additional file 1: Figure S1). Finally, to develop actively-targeted NPs, RSV/PMT-BSA-QDs NPs (F2) were reacted with d-mannose via Maillard reaction to form Schiff’s base [38]. The successfulness of the mannosylation reaction was indicated by sugar detection according to the phenol–sulfuric acid test. In this method, hexoses are dehydrated by sulfuric acid to hydroxymethyl furfural which then reacts with phenol to produce a yellow-gold color (Additional file 1: Figure S2) [39]. Further confirmation of the mannosylation reaction was revealed by the increase in the net negative charge of BSA upon coupling with mannose from − 7.0 to − 12.0 mV revealing a reduction in the number of free amino groups. In the study of Bejaars et al., the surface charge of albumin became more negative with more mannose groups conjugated to its structure [40]. Moreover, ^1^H-NMR spectra of mannose-BSA NPs revealed the characteristic peaks of mannose protons (Additional file 1: Figure S3). Mann-targeted RSV/PMT BSA-QDs NPs (F3) showed an average size of 193.9 ± 4.8 and zeta potential of − 33.1 ± 1.2 with a narrow size distribution (PDI = 0.184) (Fig. 3a). When observed by TEM, Mann-targeted RSV/PMT BSA-QDs NPs (F3) were in the size range of 95–100 nm with a spherical shape and smooth surface without any aggregation confirming high colloidal stability (Fig. 3b). The apparent size measured by TEM was slightly less than that measured by dynamic light scattering due to dehydration-induced shrinkage of particles during preparation for TEM analysis [41, 42].

### In vitro drug release

A biphasic release profile of entrapped RSV from the BSA-PMT NPs was observed. After the first 2 h, about 20.6% of RSV was released from Mann-targeted RSV/PMT BSA-QDs NPs (F3) corresponding to the surface-adsorbed drug fraction. This relatively fast release phase was followed by a sustained RSV release of about 63.4% after 24 h corresponding to the drug fraction entrapped inside the nanomatrix [43]. In comparison, free RSV was completely released after only 2 h (Fig. 3c).

In contrast, the release rate of PMT was very much slower with only 5% released from Mann-targeted RSV/PMT BSA-QDs NPs (F3) after 30 min in comparison to the rapid complete release of free PMT after 2 h (Fig. 3d). This small drug fraction released may be explained by the loss of physically bound drug associated within the hydrophobic binding sites of albumin. After 24 h, 90% of the drug was still linked to the protein. This high stability of the PMT-BSA conjugate can be ascribed to the strong amide bond between the conjugated drug and BSA. Similarly, about 95 and 91% of paclitaxel [44] and methotrexate [45], respectively remained linked to HSA after 72 h of incubation in pH 7.4 PBS indicating the stability of their conjugates. Based on these findings, it can be hypothesized that direct conjugation of PMT to the protein backbone will hinder its release in the circulation after i.v. administration of our NPs resulting in very low drug concentration; hence less side effects are expected whereas released at tumor sites after bond cleavage via lysosomal enzymes. On the contrary, when PMT was physically encapsulated into PEG–peptide–PCL NPs, an initial burst of more than 30% of the drug in the first 3 h was observed [11].

### Solid state characterization

RSV in its natural state exists as crystals, which are characterized by the melting endothermic peak around 265 °C in its thermogram (Additional file 1: Figure S4A). The thermogram of RSV-PC complex demonstrated a broad peak at 281.567 °C, indicating a successful complexation between RSV and phosphatidylcholine [46, 47]. On the other hand, PMT thermogram demonstrated three characteristic peaks at 91.784, 153.818 and 243.8 °C [48]. The endothermic peak of RSV has been disappeared in the thermograms of the non-targeted (F2) and targeted (F3) BSA NPs, suggesting that RSV was molecularly dispersed as amorphous state into the protein matrix. On the other hand, PMT endothermic peaks at 91.784 and 243.8 °C have disappeared, while its endothermic peak at 153.818 °C has been shifted to 150.1 °C with sharper intensity in the thermogram of both NPs which indicates successful conjugation between PMT and BSA, rather than existing in a free state. In the FTIR spectra of non-targeted (F2) and targeted (F3) BSA NPs, the characteristic peak of RSV at 3450–3100 cm^−1^ corresponding to its three hydroxyl groups stretching, was overlapped with the O–H stretching vibration of BSA at 3430 cm^−1^ confirming the encapsulation of RSV within BSA NPs (Additional file 1: Figure S4B). Furthermore, the carbonyl stretching peak of PMT COOH group at 1703 cm^−1^ disappeared due to the conjugation between the drug and BSA (more details in Additional file 1).

### Physical stability and redispersibility

**Fig. 4:**
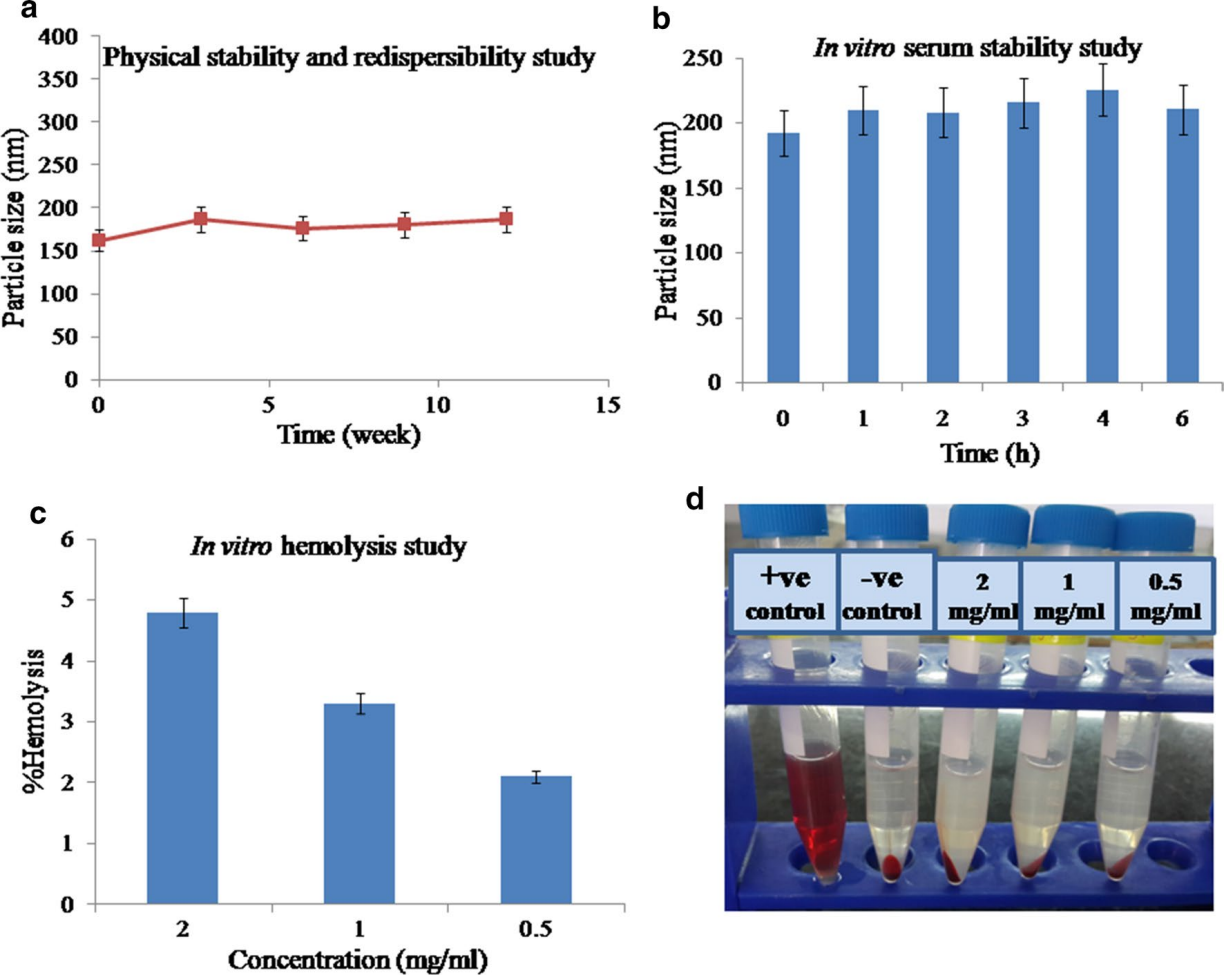
Physical stability of Mann-targeted RSV/PMT BSA-QDs NPs (F3) showing the change in particle size with time (**a**), particle size of F3 after incubation in 10% fetal bovine serum (FBS) for 6 h at 37 °C (**b**). Hemolytic potential of dual drug loaded targeted BSA-QDs NPs (F3) showing % hemolysis (**c**) and hemocompatibility images (**d**) of F3 after 1 h of incubation at 37 °C

There were no remarkable changes observed for PS and PDI of Mann-targeted RSV/PMT BSA-QDs NPs (F3) after storage at 4 °C for 3 months (Fig. 4a). The NPs showed size of 187 ± 2.3 nm and zeta potential of − 23 ± 1.2 mV after 3 months of storage, compared to the initially stored NPs (162.4 ± 3.5 nm and − 26.4 ± 0.87 mV). The results were in agreement with the high stability of paclitaxel/sorafenib co-loaded BSA NPs with no significant change in PS and zeta potential upon storage for 2 months [49].

To further enhance the storage stability of our developed NPs, they were solidified via lyophilization technique [50]. Using mannitol (5% w/v) as a cryoprotectant to facilitate the drying of NPs and prevent their aggregation, the reconstituted lyophilized Mann-targeted RSV/PMT BSA-QDs NPs (F3) demonstrated PS of 189.7 ± 6.7 nm, with acceptable redispersibility index value of 0.97 (Table 2) [51]. Similarly, doxorubicin-loaded HSA NPs were freeze-dried using mannitol as a cryoprotectant and showed insignificant size change after reconstitution [52].

**Table 2:** Effect of freeze-drying on the physicochemical characteristics of Mann-targeted RSV/PMT-BSA-QDs NPs (F3)

Formula	Yield (%w/w)	Particle size (nm)		RI*	Zeta potential (mV)	
		Before	After		Before	After
Mann-targeted RSV/PMT-BSA-QDs NPs (F3)	92.30	193.9 ± 4.8	189.7 ± 6.7	0.97	− 33.1 ± 1.2	− 24.3 ± 2.1

* *RI* redispersibility index (final particle size/initial particle size)

### In vitro hemolysis and serum stability

To further predict the feasibility of i.v. administration, the NPs stability in serum was evaluated. With addition of aqueous serum solution, Mann-targeted RSV/PMT BSA-QDs NPs (F3) showed insignificant change in the size distribution compared with the initially prepared NPs (from 192.2 ± 0.802 to 210.6 ± 0.8 nm). After 4 h of incubation with FBS, the PS of NPs (F3) reached 225 ± 3.3 nm which was decreased to 210.6 ± 1.3 nm after 6 h. This behavior could be ascribed to the association and dissociation of protein molecules on the surface of NPs during incubation period [53, 54]. The repulsive forces between the negatively charged serum proteins and BSA NPs may explain their high serum stability (Fig. 4b).

Moreover, the hemato-compatibility of Mann-targeted RSV/PMT BSA-QDs NPs (F3) in different concentration ranges (0.5–2 mg/ml) was determined and the leakage of hemoglobin from RBCs was used to quantitatively determine the membrane-damaging properties of NPs (Fig. 4c, d). At a concentration of 2 mg/ml, the NPs demonstrated 4.8% hemolysis while lower hemolysis (3.3%) was obtained at a lower NPs concentration of 1 mg/ml. This acceptable hemolytic activity of the prepared NPs could be ascribed to using biodegradable and biocompatible nanovehicles as albumin, being the major plasma protein. Moreover, albumin was reported to have a preventive effect against erythrocyte hemolysis [55].

### Cytotoxicity study

**Fig. 5:**
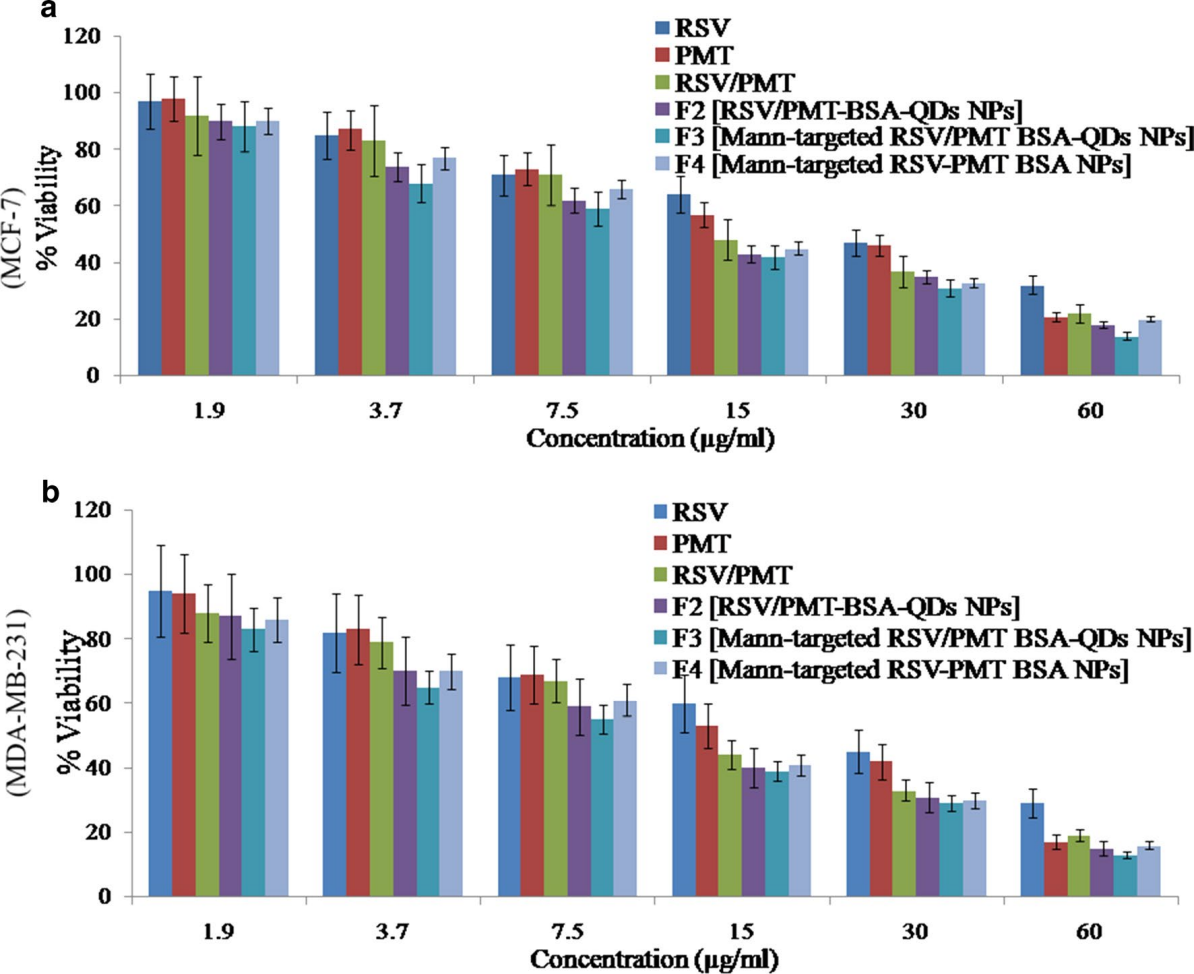
Cytotoxicity analysis showing  % cell viability of free RSV, free PMT and free RSV/PMT co-solvent compared to the prepared nano-formulations F2 (RSV/PMT-BSA-QDs NPs), F3 (Mann-targeted RSV/PMT BSA-QDs NPs), and F4 (Mann-targeted RSV-PMT BSA NPs) on MCF-7 (**a**) and MDA-MB-231 (**b**) breast cancer cell line at the concentration of 0–60 μg/ml after 24 h

RSV is a phytoestrogen with a greater effect on hormone-responsive MCF-7 breast cancer cells [56]. On the other hand, the cytotoxic drug, PMT inhibits purine and pyrimidine synthesis thus it would have a great impact on MDA-MB-231 triple negative breast cancer cells (TNBC) which are very prone to cytotoxic agents due to the lack of the DNA repairing capability [57]. Blank NPs demonstrated very little toxicity to MCF-7 and MDA-MB-231cells (viability was > 95% after 24 h). The IC_50_ of free drugs in the mixed RSV/PMT solution at 24 h was 0.5- and 0.7-fold that of RSV on MCF-7 and MDA-MB-231 cells, respectively and was 0.8-fold that of PMT on both cells. The reduction of IC_50_ values of the drugs in this combination proved synergistic cytotoxicity which is consistent with the reported synergistic cytotoxicity of RSV/PMT mixture on NSCLC cells [16]. Mann-targeted RSV/PMT BSA-QDs NPs (F3) enhanced the combination potency as demonstrated by the reduced IC_50_ on both cells compared to the free combined drug solution and the non-targeted RSV/PMT-BSA-QDs NPs (F2) (Fig. 5).

**Fig. 6:**
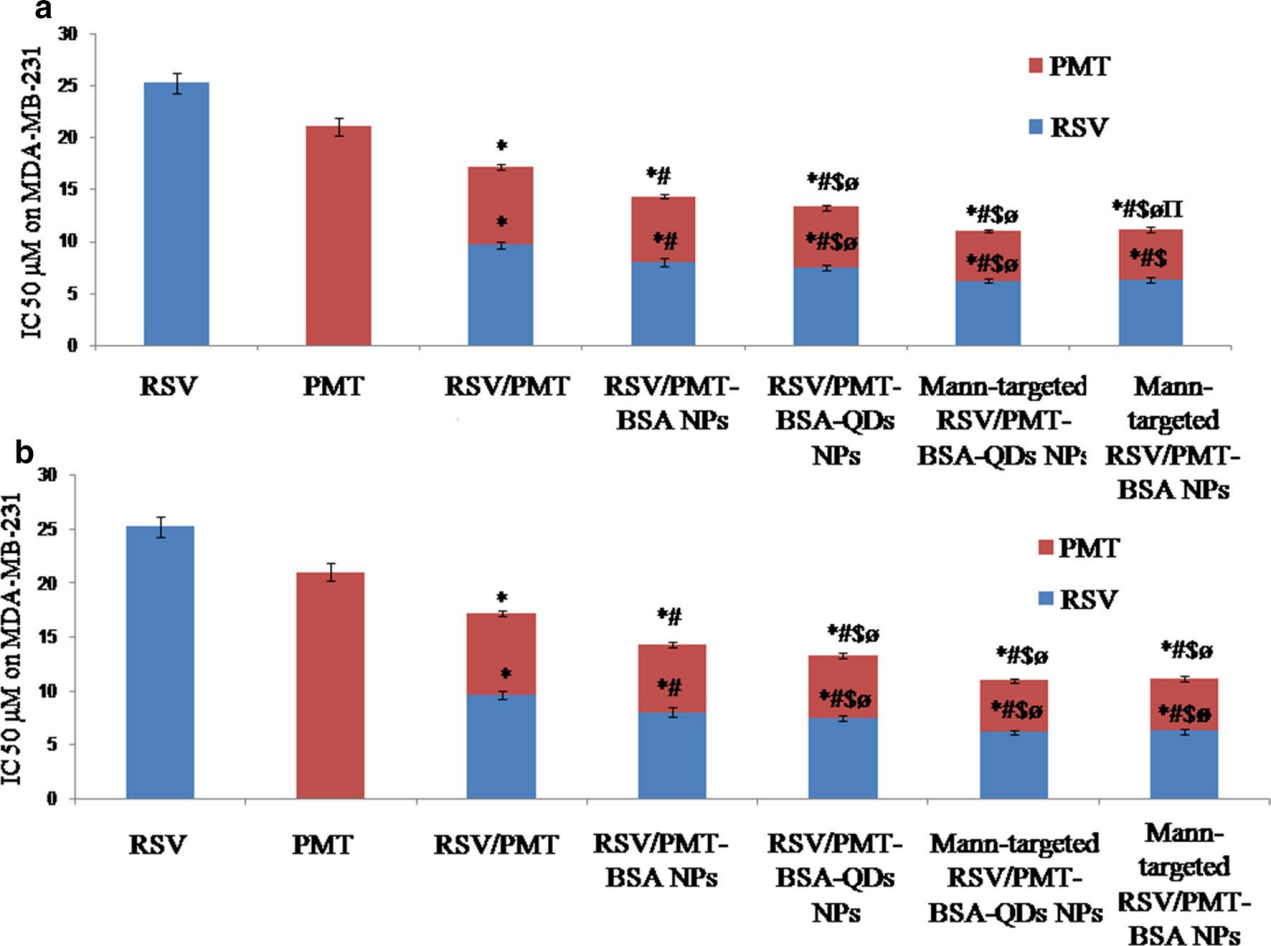
IC_50_ of free RSV, free PMT and free RSV/PMT co-solvent compared to the prepared nano-formulations F2 (RSV/PMT-BSA-QDs NPs), F3 (Mann-targeted RSV/PMT BSA-QDs NPs), and F4 (Mann-targeted RSV-PMT BSA NPs) on MCF-7 (**a**) and MDA-MB-231 (**b**) breast cancer cell line at the concentration of 0–60 μg/ml after 24 h. *p < 0.05 vs. Free drug, ^#^p < 0.05 vs. Free RSV/PMT solution, ^$^p < 0.05 vs. the drug in F1, ^ø^p < 0.05 vs. the drug in F2, ^π^p < 0.05 vs. the drug in F3

Further statistical analysis was done using CompuSyn software (version 1) described by *Chou and Talalay* where we used Combination Index (CI) and Dose Reduction Index (DRI) in comparing the different NPs with free drug combination (Fig. 6) [58]. The obtained results ensure the superiority of different drug loaded NPs compared to the free combination, especially Mann-targeted RSV/PMT BSA-QDs NPs (F3) where its CI was 0.813 and 0.873 in MCF-7 and MDA-MB-231 cells, respectively, revealing that these NPs succeeded in achieving synergy between RSV and PMT. Moreover, the DRIs of RSV were 4.3 and 4.08 for MCF-7 and MDA-MB-231 cells, respectively in Mann-targeted RSV/PMT BSA-QDs NPs (F3). While the DRIs of PMT were 4.25 and 4.35 for MCF-7 and MDA-MB-231cells, respectively in Mann-targeted RSV/PMT BSA-QDs NPs (F3). The superior anti-cancer efficacy of mannose-targeted NPs (F3) could be attributed to their higher internalization into breast cancer cells via mannose-receptor mediated endocytosis (Tables 3, 4) [59]. The lower IC_50_ of BSA-QDs NPs (F2 and F3) than QDs-free BSA NPs F4) may be attributed to ROS generated by QDs which may cause cell apoptosis. A greater cell growth inhibition capability of QDs was also reported by Zhao et al. against HepG2 hepatocellular carcinoma and HeLa cells with 11-fold lower cytotoxicity compared to QSG-7701 human hepatocytes. This preferential killing of cancer cells by QDs can be improved by using more selective targeting ligands which was achieved in our approach using mannose-targeted BSA-QDs NPs [3]. Thus, the nanotoxicity of QDs can be potentially converted to a new therapeutic option.

**Table 3:** IC_50_, Combination Index (CI) and Dose Reduction index (DRI) values of free RSV, free PMT and free RSV/PMT co-solvent compared to the prepared nano-formulations on MCF-7 breast cancer cell line at the concentration of 0–60 μg/ml after 24 h

Drug/combo	CI value	Total IC_50_ of combination	Dose RSV	Dose PMT	DRI of RSV	DRI of PMT
Free RSV	–	–	22.24	–	–	–
Free PMT	–	–	–	17.21	–	–
Blank NPs	–	–	–	–	–	10,739
RSV/PMT	0.981	13.95	7.86	6.15	2.82	2.79
F1 [RSV/PMT-BSA NPs]	0.926	11.1	6.26	4.89	3.55	3.51
F2 [RSV/PMT-BSA-QDs NPs]	0.909	10.96	6.18	4.83	3.59	3.56
F3 [Mann-targeted RSV/PMT-BSA-QDs NPs]	0.813	9.17	5.17	4.04	4.30	4.25
F4 [Mann-targeted RSV/PMT-BSA NPs]	0.901	10.1	5.69	4.45	3.90	3.86

**Table 4:** IC_50_, Combination Index (CI) and Dose Reduction index (DRI) values of free RSV, free PMT and free RSV/PMT co-solvent compared to the prepared nano-formulations on MDA-MB-231 breast cancer cell line at the concentration of 0–60 μg/ml after 24 h

Drug/combo	CI value	Total IC_50_ of combination	Dose RSV	Dose PMT	DRI of RSV	DRI of PMT
RSV	–	–	25.28	–	–	–
PMT	–	–	–	21.06	–	–
Blank NPs	–	–	–	–	–	28,128
RSV/PMT	1.04	17.11	9.65	7.54	2.61	2.79
F1 [RSV/PMT-BSA NPs]	0.975	14.25	8.03	6.28	3.14	3.35
F2 [RSV/PMT-BSA-QDs NPs]	0.931	13.24	7.46	5.83	3.38	3.60
F3 [Mann-targeted RSV/PMT-BSA-QDs NPs]	0.873	10.96	6.18	4.83	4.08	4.35
F4 [Mann-targeted RSV/PMT-BSA NPs]	0.910	11.13	6.27	4.90	4.02	4.29

### Intracellular-uptake of NPs

**Fig. 7:**
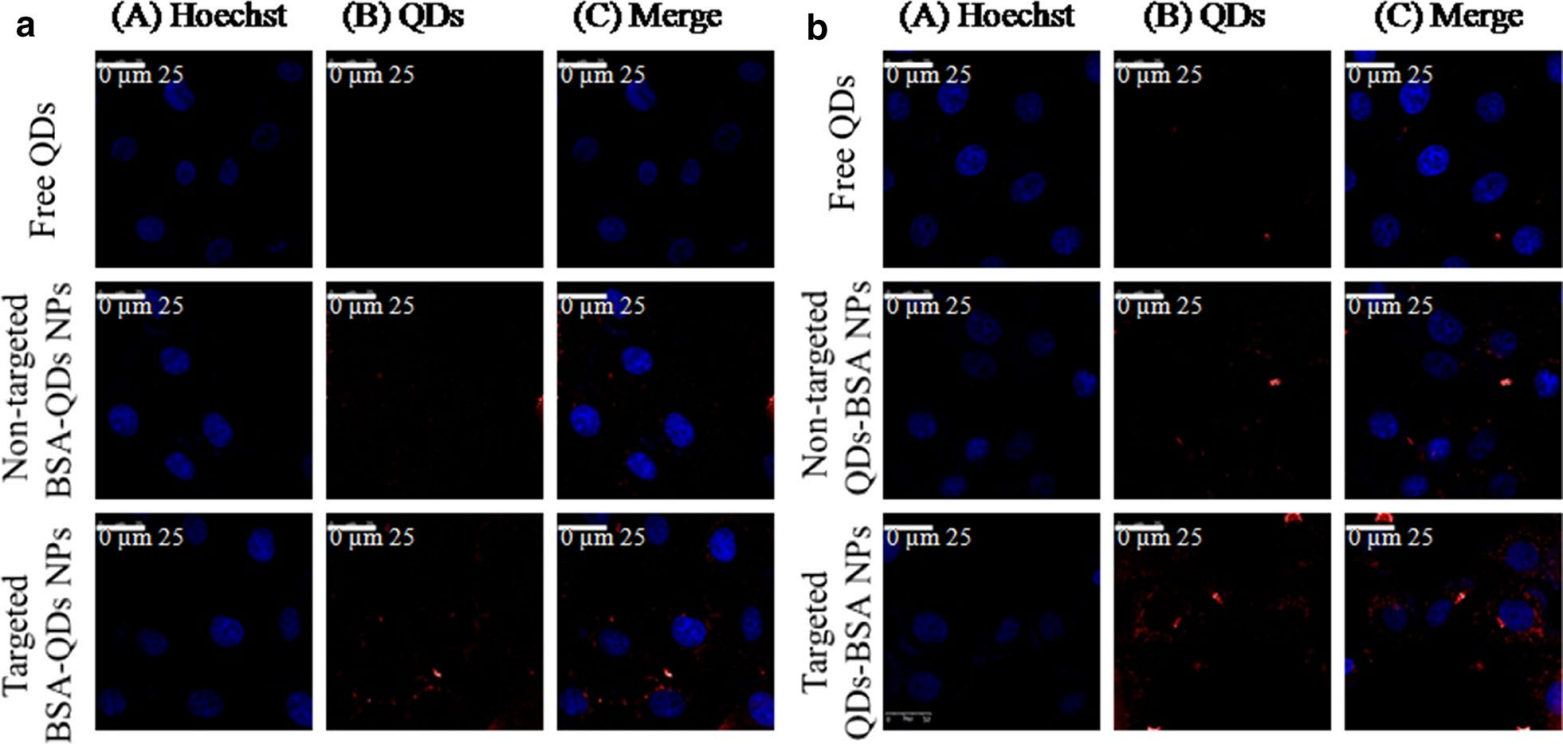
Confocal images showing cellular uptake of QDs, non-targeted BSA-QDs NPs and targeted BSA-QDs NPs within MCF-7 breast cancer cells after incubation for 4 h (**a**) and 24 h (**b**)

The cellular internalization capacity of Mannose-targeted BSA-QDs NPs, non-targeted BSA-QDs NP sand free QDs by MCF-7 breast cancer cells (over-expressing mannose receptors on their surface) was visualized via fluorescent images obtained by confocal laser scanning microscopy and the images were analyzed using Image J software (Fig. 7). The targeted NPs demonstrated higher internalization into the cancer cells in comparison with non-targeted ones as indicated by the strong red fluorescence intensity observed in cells treated with mannose-targeted NPs. On the other hand, weak red fluorescence intensity was demonstrated in the cells incubated with free QDs. After 24 h incubation, the fluorescence intensity for both non-targeted and targeted NPs increased, while free QDs showed the lowest fluorescence intensity. Mannose-functionalized BSA NPs can interact with mannose receptors resulting in enhanced internalization into cancer cells over-expressing those receptors via endocytosis. Moreover, the uptake of mannose-capped silicon NPs within MCF-7 breast cancer cells was found to be faster than non-functionalized ones. In addition to the role of mannose, albumin-based NPs have been reported to improve accumulation of drugs into tumor cells through interaction with the albumin binding receptors SPARC (secreted protein acidic and rich in cysteine) and albondin (glycoprotein 60) overexpressed on tumor and vascular cells [30].

### Tumor localization of NPs

**Fig. 8:**
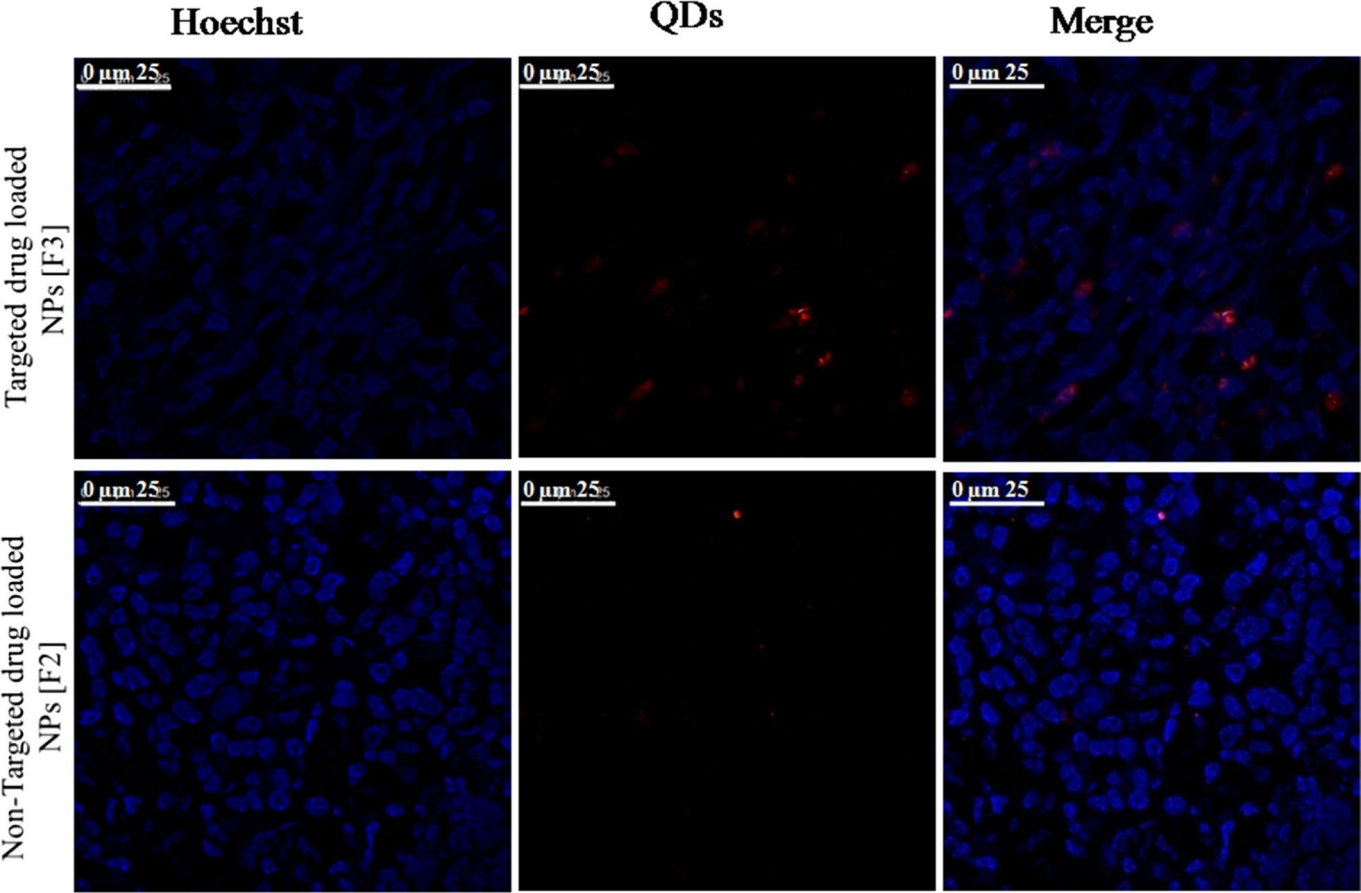
Mann-targeted RSV/PMT BSA-QDs NPs (F3), non-targeted RSV/PMT-BSA-QDs NPs (F2) displayed fluorescence in breast tumor tissues frozen slices after administration to Ehrlich-Induce mammary tumor in mice

To trace the NPs accumulation within tumor tissue, the fluorescence of the harvested tumor was visualized via confocal microscope imaging. Higher fluorescence intensity was detected in the tumor tissues of mice groups treated with Mann-targeted RSV/PMT BSA-QDs NPs (F3) compared to non-targeted RSV/PMT-BSA-QDs NPs (F2) which ensures their effective localization in the tumor tissues (Fig. 8). Thus, active targeting with mannose has enhanced the tumor accumulation of NPs in comparison with non-targeted ones. The strong fluorescence of QDs enabled imaging of the NPs accumulated in tumor tissue thus confirming the suitability of our NPs for theranostic applications.

### In vivo anti-tumor efficacy

**Fig. 9:**
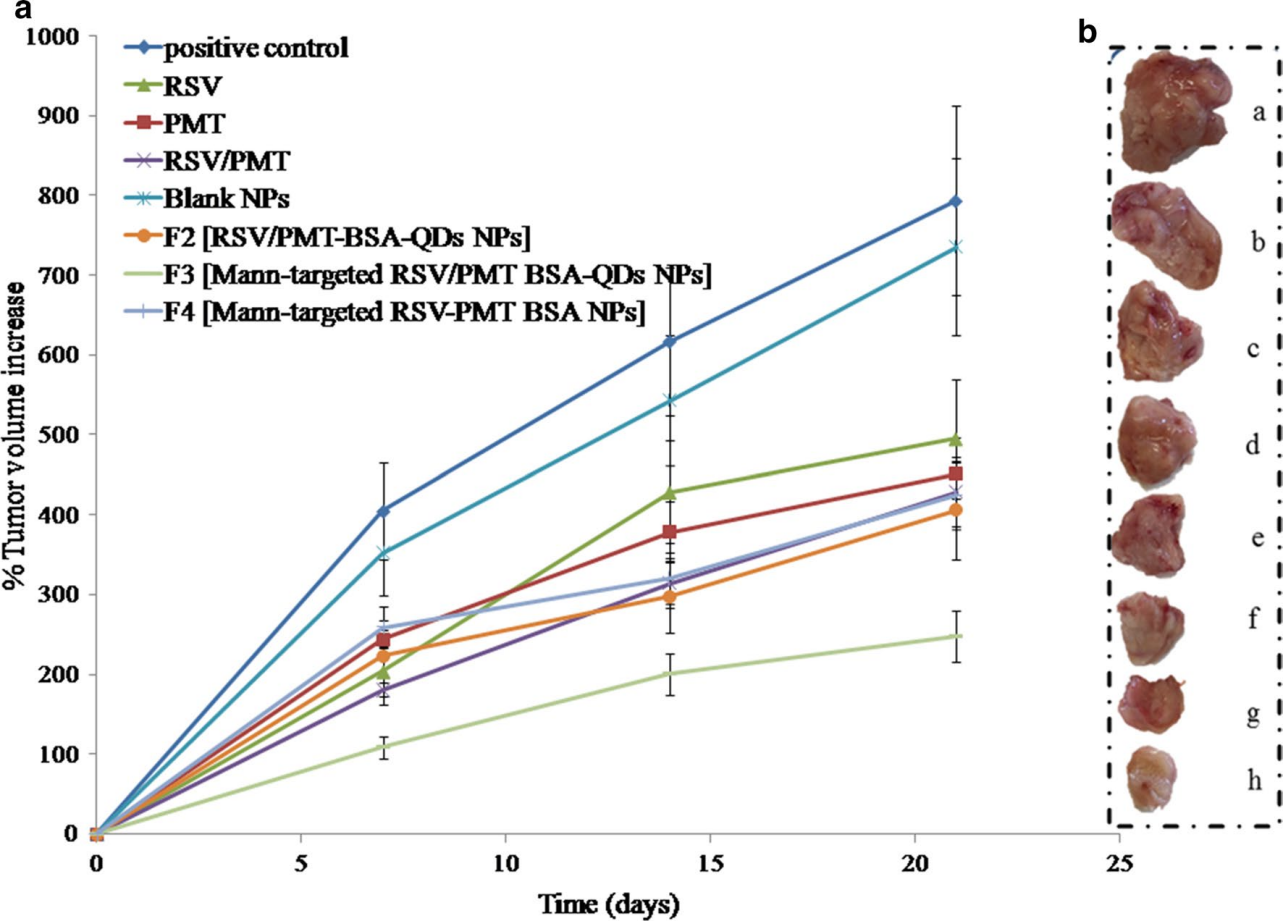
In-vivo anti-tumor efficacy showing percentage increase in volume of Ehrlich-induce mammary tumor of mice at indicated time-points along the experiment duration (**a**) and representative photo of the excised tumor from each group at the end of the study period (**b**)

The in vivo antitumor activity of the dual-drug loaded BSA NPs was further investigated on Ehrlich ascites mammary tumor bearing mice. At the end of study, the un-treated positive control group had demonstrated the highest and uncontrollable % increase of tumor volume corresponding to 793.98%. Co-administration of free RSV/PMT co-solvent system succeeded in limiting the  % increase of tumor volume to 429% which is higher than each drug alone. On the other direction, the developed NPs demonstrated a significant (p < 0.05) remarkable reduction of tumor growth in comparison with the positive control and free drug-treated groups. The most powerful suppression of tumor growth was demonstrated by Mann-targeted RSV/PMT BSA-QDs NPs (F3) showing 247% increase in tumor volume, respectively compared to 405% for non-targeted RSV/PMT-BSA-QDs NPs (F2). Moreover, it is worthy to note that coupling of QDs to BSA NPs was found to exert an additional suppressive effect on tumor growth besides their imaging capability. Mann-targeted RSV/PMT BSA-QDs NPs (F3) displayed 247% increase in tumor volume which was significantly (p < 0.05) lower than its counterpart NPs without QDs (Mann-targeted RSV-PMT BSA NPs, F4) with 425% increase in tumor volume (Fig. 9a, b). The in vivo therapeutic action of QDs was previously proved using hepatocellular carcinoma animal model where Zhao et al. prepared polyamine-coated CdSe/ZnS QDs and tested its therapeutic effect on hepatocellular carcinoma Hep G2, and hepatocyte QSG-7701 cells. It was found that the IC_50_ values of QDs at 48 h are 2.51 and 26.65 µM for HepG2 and QSG-7701 cells, respectively. This approximately 11-fold difference reflected the preferential cell killing capabilities of QDs between the two cell lines, a desired property that can be further improved by using more selective targeting ligands. Several lines of evidence suggest that the antitumor effect of QDs rises from ROS-induced cell apoptosis. In vivo, the mean survival time of tumor-bearing mice could be extended by 2.5 times when treated with QDs. These results demonstrated the possibility of converting nano-toxicity of QDs to antitumor activity [3].

Moreover, animals’ body weight changes (as an important pointer for animal health) were recorded during the study period. It was observed that mice treated with free drugs appeared weak and demonstrated a reduction in their body weight after treatment while no observable bodyweight loss was noticed in mice groups treated with drug-loaded NPs (F2 and F3). Thus, the prepared NPs succeeded to reduce the toxicity of free drugs throughout experimental period (Details of the body weight measurements are in Additional file 1: Table S1, Figure S5.

**Fig. 10:**
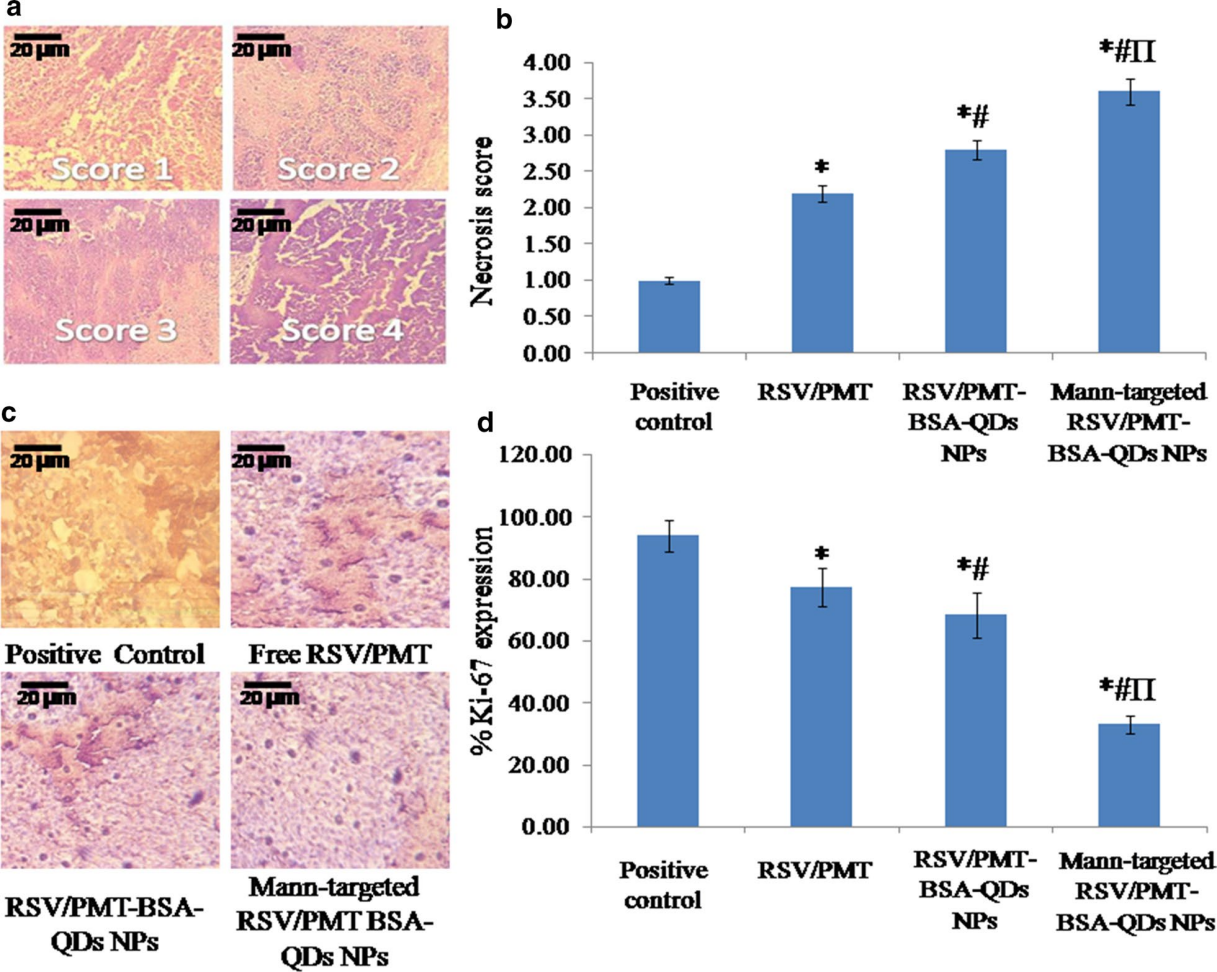
Scoring of necrosis of Ehrlich-induce mammary tumor in mice (**a**) and necrotic score of Ehrlich-induce mammary tumor in free RSV/PMT solution, Mann-targeted RSV/PMT BSA-QDs NPs (F3) and non-targeted RSV/PMT-BSA-QDs NPs (F2)-treated mice group compared to positive control group (**b**). Immunohistopathological staining of the proliferative marker Ki-67 in breast cancer tissues of positive control group and free RSV/PMT combination, non-targeted F2 and targeted F3-mice treated group (**c**), % Ki-67 proliferation marker in positive control group and breast cancer tissues of free RSV/PMT combination, non-targeted F2 and targeted F3- mice treated group (**d**). *p < 0.05 vs. positive control, ^#^p < 0.05 vs. free RSV + PMT, ^Π^p < 0.05 vs. F2 (non-targeted NPs)

Figure 10a, b showed scoring of necrosis of Ehrlich-induced mammary tumor in mice. The targeted and non-targeted drug-loaded NPs (F3 and F2, respectively) showed significantly (p < 0.05) higher necrosis compared to the positive control group thus confirming their efficacy. Furthermore, immunohistochemical analysis revealed a significantly (p < 0.05) reduced staining density of the proliferation protein Ki-67 in the tumor samples of mice treated with targeted and non-targeted NPs (F3 and F2, respectively) (32.9 and 68.31% Ki-67 expression, respectively) compared to positive control groups (93.92% Ki-67 expression) (Fig. 10c, d). This reflected the particularly high suppressive power of NPs on proliferation of tumor cells thereby inhibiting the tumor growth [60].

**Fig. 11:**
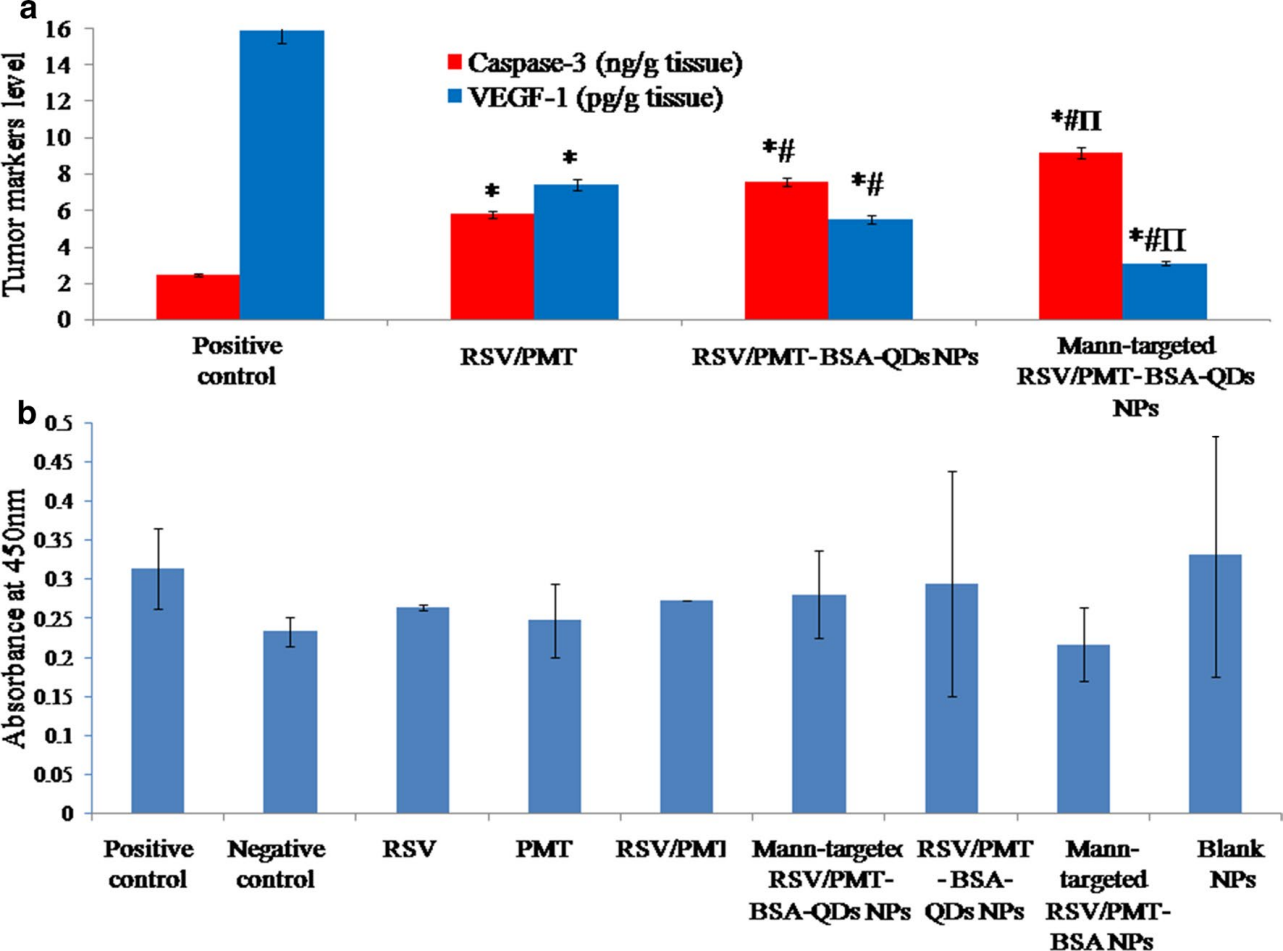
Comparison between the studied groups [free RSV/PMT co-solvent Mann-targeted RSV/PMT BSA-QDs NPs (F3) and non-targeted RSV/PMT-BSA-QDs NPs (F2)-mice treated groups] in addition to the positive control group according to active caspase-3 and VEGF-1 levels (**a**). *p < 0.05 vs. positive control, ^#^p < 0.05 vs. free RSV + PMT, ^Π^p < 0.05 vs. F2 (non-targeted NPs). Assessment of immunogenicity of the BSA nano-delivery systems (**b**). No significant difference was found in immunogenic response between the treated groups and the control group (p > 0.05 by ANOVA test and Tukey–Kramer post hoc multiple comparison test)

Relative to the positive control group, the targeted and non-targeted dual drug-loaded NPs (F3 and F2, respectively) exhibited a significantly higher (p < 0.05) apoptotic effect with 3.724- and 3.07-folds increase in Caspase-3 level, respectively versus only 2.35-folds elevation for free RSV/PMT combination (Fig. 11a) [14]. Moreover, the targeted and non-targeted NPs (F3 and F2, respectively) also succeeded to significantly (p < 0.05) reduce the level of angiogenic factor VEGF-1 by 5.125- and 2.883-folds, respectively versus 2.127-folds reduction for free RSV/PMT combination as compared to the positive control (Fig. 11a) [28].

### Immunogenicity of the BSA nano-delivery system

One of the concerns with the use of protein-based NPs is the risk of immunogenicity when administered in vivo. Many studies have used albumin-based NPs by i.v. injections and no immunogenic or antigenic response to albumin NPs was reported [30]. In our study, no significant difference in the level of anti-BSA IgG antibodies was observed between any of the treated groups and the non-treated group. This demonstrates that under the experimental conditions of the animal trial, the BSA delivery system was not immunogenic (Fig. 11b).

From all the above mentioned results, it is clear that the anti-tumor efficacy of PMT has been enhanced in a multi-step approach including: (a) co-administration of RSV with PMT, may maximize the therapeutic effect of both drugs via modulating different signaling pathways thus leading to higher anti-tumor efficacy, reduced toxicity and may also overcome the multi-drug resistance, (b) incorporation of the drug combination into BSA NPs, to benefit from albumin-mediated accumulation of drugs into tumor tissue via binding to albondin and SPARC, the small NP size-mediated EPR effect as well as the sustained drug release profile. Specifically, covalent attachment of PMT to albumin structure hindered its release into circulation and enabled tumor-specific drug release, (c) active targeting of the dual drug-loaded NPs via mannose coupling imparted the ability to interact with mannose receptors overexpressed on the surface of breast cancer cells allowing more internalization of NPs and hence better drug accumulation in tumor cells, (d) incorporation of QDs by conjugation to NPs surface not only enabled imaging of the tumor for theranostic applications and revealing NPs tissue distribution but also contributed to enhance the anti-tumor efficacy may be via ROS generation mechanism. Moreover, covalent coupling rather than physical encapsulation enables tumor-specific release of QDs thus reducing their systemic toxicity.

## Conclusion

In summary, we designed a multifunctional nanoplatform of mannose-coupled BSA-QD NPs for targeted synergistic co-delivery of PMT and RSV as well as to enable a fluorescence-based imaging of breast cancer cells. The formulated dual drug-loaded nanocarriers demonstrated optimal physicochemical properties of small size, high drug loading, retarded drug release, low hemolytic activity, good colloidal and serum stability as well as non-immunogenicity. This nanoplatform could be successfully internalized by breast cancer cells resulting in enhanced cytotoxicity. In addition, systemic delivery of this nanoplatform remarkably reduced the tumor volume in vivo. Finally, this work provides a new theranostic platform of mannosylated albumin–QD nanohybrids for targeted co-delivery of PMT and RSV to breast cancer cells.

## Additional file

**Additional file 1.** The additional information file include Characterization of the synthesized CdTe QDs, Preparation of PMT-conjugated BSA (PMT-BSA), Preparation of RSV-PC complex, Solid state characterization including FTIR Spectroscopy and DSC Thermograms, methodology of physicochemical characterization of dual drug-loaded BSA NPs including Drug loading and encapsulation efficiency, Particle size and zeta potential analysis, In vitro drug release, Physical stability study, Freeze drying and redispersibility, Morphological analysis, In vitro hemolysis and serum stability, In vitro cytotoxicity, Cellular-uptake study, the methodology of In vivo studies including Tumor growth biomarkers, Histopathological analysis, Immunohistochemical analysis, Quantification of the proliferative marker Ki-67 by image analysis technique and tissue localization of NPs. Immunogenicity of the nano-delivery system, Statistical analysis, the analysis of the FTIR study and body weight measurement study and the table of body weight average of mice groups. It also includes five figures which are: **Figure S1.** Physicochemical properties of the prepared RSV/PMT-BSA-QDs NPs (F2); size distribution diagram of non-targeted drug loaded BSA-QDs NPs (F2) (A) and their corresponding zeta potential distribution (B). **Figure S2.** A photograph illustrating the phenol sulfuric acid test of BSA (A) and Mannose-BSA NPs conjugate (B). **Figure S3.** ^1^H-NMR spectra of mannose-BSA, BSA and mannose revealing the presence of mannose protons in the spectra of mannose-BSA conjugate. **Figure S4.** DSC thermogram of RSV, RSV-PC complex, PMT, RSV/PMT-BSA-QDs NPs (F2) andMann-targeted RSV/PMT BSA-QDs NPs (F3) (A) and Fourier Transform Infrared (FTIR) spectra of RSV, RSV-PC complex, PMT, F2 and F3 (B). **Figure S5.** In-vivo anti-tumor efficacy showing change in body weights measurements of mice at indicated time-points along the experiment duration (C).

## Abbreviations

BSA NPs: bovine serum albumin nanoparticles
CLRs: C-type lectin receptors
CI: Combination Index
DSC: differential scanning calorimetry
DRI: Dose Reduction Index
DLS: dynamic light scattering microscope
EAT: Ehrlich ascites tumor
ELISA: enzyme linked immunosorbent assay
EPR: enhanced permeability and retention
FBS: fetal bovine serum
HPLC: high performance liquid chromatography
IC_50_: inhibitory concentration
MR: mannose receptor
MDR: multi-drug resistance
NMR: nuclear magnetic resonance
PMT: pemetrexed
PBS: phosphate buffer saline
PC: phosphatidylcholine
PL: photoluminescence
PDI: polydispersity index
QDs: quantum dots
ROS: reactive oxygen species
RSV: resveratrol
RES: reticuloendothelial system
SPARC: secreted protein acidic and rich cysteine
TEM: transmission electron microscope
TNBC: triple negative breast cancer
VEGF-1: vascular endothelial growth factor
