# Supplementary material for: Combining hydrophilic chemotherapy and hydrophobic phytotherapy via tumor-targeted albumin–QDs nano-hybrids: covalent coupling and phospholipid complexation approaches
Source: J Nanobiotechnology. 2019 Jan 19;17:7. doi: 10.1186/s12951-019-0445-7 (PMC6339697; doi:10.1186/s12951-019-0445-7)
Supplement: Supplementary file 1 — Additional file 1. The additional information file include Characterization of the synthesized CdTe QDs, Preparation of PMT-conjugated BSA (PMT-BSA), Preparation of RSV-PC complex, Solid state characterization including FTIR Spectroscopy and DSC Thermograms, methodology of physicochemical characterization of dual drug-loaded BSA NPs including Drug loading and encapsulation efficiency, Particle size and zeta potential analysis, In vitro drug release, Physical stability study, Freeze drying and redispersibility, Morphological analysis, In vitro hemolysis and serum stability, In vitro cytotoxicity, Cellular-uptake study, the methodology of In vivo studies including Tumor growth biomarkers, Histopathological analysis, Immunohistochemical analysis, Quantification of the proliferative marker Ki-67 by image analysis technique and tissue localization of NPs. Immunogenicity of the nano-delivery system, Statistical analysis, the analysis of the FTIR study and body weight measurement study and the table of body weight average of mice groups. It also includes five figures which are: Figure S1. Physicochemical properties of the prepared RSV/PMT-BSA-QDs NPs (F2); size distribution diagram of non-targeted drug loaded BSA-QDs NPs (F2) (A) and their corresponding zeta potential distribution (B). Figure S2. A photograph illustrating the phenol sulfuric acid test of BSA (A) and Mannose-BSA NPs conjugate (B). Figure S3. 1H-NMR spectra of mannose-BSA, BSA and mannose revealing the presence of mannose protons in the spectra of mannose-BSA conjugate. Figure S4. DSC thermogram of RSV, RSV-PC complex, PMT, RSV/PMT-BSA-QDs NPs (F2) andMann-targeted RSV/PMT BSA-QDs NPs (F3) (A) and Fourier Transform Infrared (FTIR) spectra of RSV, RSV-PC complex, PMT, F2 and F3 (B). Figure S5. In-vivo anti-tumor efficacy showing change in body weights measurements of mice at indicated time-points along the experiment duration (C). [file 12951_2019_445_MOESM1_ESM.docx]

**Additional Information**

**Combining hydrophilic chemotherapy and hydrophobic phytotherapy via tumor-targeted albumin-QDs nano-hybrids: Covalent coupling & phospholipid complexation approaches**

Dina G. Zayed^a,b^, Shaker M. Ebrahim^c^, Maged W. Helmy^d^, Sherine N. Khattab^a,e^, Mohammed Bahey-El-Din^f^, Jia-You Fang^g,h,i*^, Kadria A. Elkhodairy^a,b^, Ahmed O. Elzoghby^a,b,j,k*^

^a^ Cancer Nanotechnology Research Laboratory (CNRL), Faculty of Pharmacy, Alexandria University, Alexandria 21521, Egypt.

^b^ Department of Industrial Pharmacy, Faculty of Pharmacy, Alexandria University, Alexandria 21521, Egypt.

^c^ Department of Materials Science, Institute of Graduate Studies and Research, Alexandria University, Alexandria 21526, Egypt.

^d^Department of Pharmacology and Toxicology, Faculty of Pharmacy, Damanhour University, Damanhour, Egypt.

^e^Department of Chemistry, Faculty of Science, Alexandria University, Alexandria 21321, Egypt.

^f^Department of Microbiology and Immunology, Faculty of Pharmacy, Alexandria University, Alexandria 21521, Egypt.

^g^ Pharmaceutics Laboratory, Graduate Institute of Natural Products, Chang Gung University, Taoyuan 333, Taiwan

^h^ Research Center for Industry of Human Ecology and Research Center for Chinese Herbal Medicine, Chang Gung University of Science and Technology, Kweishan, Taoyuan 333, Taiwan

^i^ Department of Anesthesiology, Chang Gung Memorial Hospital, Kweishan, Taoyuan 333, Taiwan

^j^ Division of Engineering in Medicine, Brigham and Women's Hospital, Department of Medicine, Harvard Medical School, Boston, MA 02115, USA

^k^ Harvard-MIT Division of Health Sciences and Technology, Cambridge, MA 02139, USA

**Experimental**

1. **Characterization of the synthesized CdTe QDs**

TEM images of the CdTe QDs were obtained by using Jeol JEM-2100 transmission electron microscope (Tokyo, Japan) by immersing copper grid in the QDs solution for 10 s followed by a drying stage. The zeta potential of the CdTe QDs was estimated by NanoZS/ZEN3600 Zetasizer (Malvern Instruments Ltd., UK). The zeta potential of diluted sample was calculated from the mean electrophoretic mobility.

1. **Preparation of PMT-conjugated BSA (PMT-BSA)**

PMT–BSA conjugate was prepared by conjugating BSA with PMT via amide bond using carbodiimide coupling reaction [[1](#_ENREF_1)]. PMT (0.014 g, 0.03mmol) was preactivated with EDC.HCl (0.014 g,0.07 mmol) and NHS (0.09 g,0.07 mmol) in 4 ml distilled water for 30 min at room temperature under stirring to ensure complete activation of carboxylate groups of PMT. Then, 100 mg of BSA were dissolved in 2 ml distilled water, added to the activated PMT solution and left for 24 h under constant stirring. The solution was then purified by dialysis against deionized water for 24 h, with water being replaced every 3–5 h to remove un-reacted PMT and finally lyophilized using lyoQuest lyophilizer (Telstar, Spain) at a pressure of 40 mbar for 48 h. The freeze-dried powder was then stored in desiccator at a shelf temperature.

1. **Preparation of RSV-PC complex**

RSV-PC complex was prepared via freeze-drying technique [[2](#_ENREF_2)] where RSV and lipoid S75 in 1:2 molar ratio were dissolved in 8 ml TBA followed by stirring for 24 h under dark conditions to allow complex formation. 250 mg mannitol was dissolved in 2 ml H_2_O and added dropwise to the complex solution just before lyophilization. The vials were lyophilized and then the freeze-dried powder was stored in desiccator at a shelf temperature. For determination of drug content of RSV-PC complex, 10 mg of RSV-PC complex was dissolved in 10 ml ethanol. The above solution was analyzed by UV spectrophotometer at 306 nm. The above procedure was repeated three times to ensure the homogeneity of the complex.

1. **Solid state characterization**

**4.1. FTIR Spectroscopy**

The FTIR spectra of pure drugs and drug-loaded nanocarriers were obtained via FTIR spectrometer (spectrum RXI, Perkin Elmer, USA). Samples were finely ground with infra-red grade dry potassium bromide then pressed into pellets. The spectra were recorded in the transmission range of 4000 to 450 cm^-1^ at room temperature [[3](#_ENREF_3)].

**4.2. DSC Thermograms**

DSC thermograms were recorded for free drugs and drug-loaded nanocarriers using a DSC 6 differential scanning calorimeter (Perkin Elmer, USA). Each sample (5 mg) was weighed precisely, placed onto flat-bottomed aluminum pan and scanned between 50-400°C with a constant heating rate of 10°C/min in presence of nitrogen atmosphere (flow rate 20 ml/min) [[4](#_ENREF_4)].

1. **Physicochemical characterization of dual drug-loaded BSANPs**

**5.1. Drug loading and encapsulation efficiency**

The **encapsulation efficiency** of RSV and the amount of conjugated PMT in the NPs were determined indirectly from the difference between the amount of free drug in the supernatant to calculate the free RSV and in the dialysate to calculate the free PMT and the total added drug amount. After each centrifugation step, the supernatants were obtained and analyzed for the drug using HPLC.

An **HPLC method** was developed for simultaneous determination of RSV and PMT. An Agilent 1260 Infinity HPLC system equipped with a quaternary pump, an autosampler, DAD (Diode Array Detector), and an Agilent Chemstation data processing system were used for the analysis. The HPLC analysis was carried out with an Agilent Zorbax Eclipse XDB C_18_ reversed-phase column (250×4.6 mm, 5 µm particle size) maintained at room temperature. For chromatographic elution, the flow rate was 1.5 ml/min over the entire separation and the injection volume was 20 µl. A step gradient method was utilized for elution in which the mobile phase consisted initially of 15% acetonitrile and 85% PBS (10 mM Na_2_HPO_4_, adjusted at pH 5.0 using orthophosphoric acid) for the first 6 min, followed by 50% acetonitrile and 50% PBS for the next 4 min. Total run time was 10 min and RSV was detected at 306 nm, while PMT was detected at 225 nm.

A good linearity was shown by the calibration curve obtained from 1 to 20 μg/ml of PMT and RSV concentration. A coefficient of determination (R^2^) of 0.999 was obtained for both RSV and PMT according to the linear regression analysis of the data (peak area versus concentration). This demonstrated that RSV and PMT peaks area using the developed methodology responded linearly to the increase in concentration within the range examined. The slope was found to be 1061±0.25 and 289±0.241 for RSV and PMT, respectively. Symmetric one separate peak corresponding to RSV was obtained at 306 nm and another separate peak for PMT was obtained at λmax 225 nm. PMT and RSV were eluted at 4.42 ±0.14 min and 11.82±0.2 min, respectively with a total analytical run time of 12 min. Selectivity was demonstrated showing that PMT and RSV peaks were free of interference from the excipients and materials used to prepare the formulations used in this study. Encapsulation Efficiency (%EE) and percentage conjugation (% conjugation) were calculated using the following equation:

%EE/ % conjugation = $\frac{Total drug-Free drug}{Total drug}\times100$ (1)

**5.2. Particle size and zeta potential analysis**

Photon correlation spectroscopy (PCS) was used for determination of **particle size (PS) and polydispersity index (PDI)** of NPs via a NanoZS/ZEN3600 Zetasizer. The PS was measured with the non-invasive backscattering technology at a detection angle of 173° after dilution with purified water to an appropriate concentration [[5](#_ENREF_5)]. All of the DLS measurements were carried out at 25°C for three repeated measurements. For the zeta potential measurement, diluted sample was placed in a universal folded capillary cell equipped with platinum electrodes. The zeta potential values were calculated from the mean electrophoretic mobility, as determined by Laser Doppler Anemometry (LDA).

**5.3. *In vitro* drug release**

**The *in vitro* release** of RSV and PMT from the dual drug-loaded BSA NPs and the free drug solution was investigated using dialysis membrane method [[6](#_ENREF_6)]. 2 ml of NPs colloidal suspension (eq. to 4.5 mg PMT and 4 mg RSV) was transferred into dialysis bags (12–14 kDa MWCO VISKING dialysis tubing, SERVA, Germany). The bags were suspended in 100 ml of phosphate buffered saline (PBS pH 7.4) and maintained at 37ºC under dark conditions in a shaking water bath at 100 rpm. Samples of 2 ml of the release medium were taken at designated time intervals and replaced with an equal volume of release medium. All samples were run in triplicates and filtered through a 0.45 µm membrane filter, and the amount of RSV and PMT released was analyzed by HPLC.

**5.4. Physical stability study**

Aliquots of the dual drug-loaded NPs suspension were stored in sealed tubes at 4°C. PS, PDI and zeta potential of the NPs were monitored at specified time points for 3 months.

**5.5. Freeze drying and redispersibility**

An aliquot of 2 ml of the NPs suspension containing mannitol (5% w/v) as cryoprotectant was placed into glass vials and frozen at -80°C then lyophilized for 24 h. The lyophilized NPs were stored in a desiccator at 25°C. For reconstitution, 2 ml of ultrapure water was added to the lyophilized NP cake followed by gentle agitation. The reconstituted NPs were assessed for PS, PDI and zeta potential. Moreover, the redispersibility index and the lyophilization yield were calculated for all formulations.

$Freeze drying yield \left( \%\frac{w}{w} \right)= \frac{The weights of the lyophilized powders collected}{The total initial mass of solids in the preparation}$(2)

$Redispersibility index \left( \mathrm{RI} \right)= Sf/\mathrm{Si}$(4)

Where Sf/Si is the ratio between particle size after lyophilization and initial one.

**5.6. Morphological analysis**

The morphology of the dual drug-loaded NPs was analyzed using transmission electron microscope (TEM) at an accelerating voltage of 80 kV. The sample was diluted with water, and a drop was then placed on a copper grid. A filter paper was used to remove the excess solution. Samples were subsequently stained with uranyl acetate solution for 30 s and then dried before examination.

**5.7. *In vitro* hemolysis and serum stability**

Rat blood samples were withdrawn from retro-orbital plexus into test tubes containing ethylene diamine tetra-acetic acid (EDTA), centrifuged and washed with saline. The obtained RBCs (1 ml) were diluted with saline to 10 ml. 2 ml of the RBCs suspension was incubated with 2 ml of the NPs suspension (1 mg/ml) at 37^o^C with gentle shaking. After 1 h, samples were separated via centrifugation at 3000 rpm for 5 min. Hemolytic activity was assessed by quantifying the amount of hemoglobin released from RBCs. The absorbance (A) of the supernatant was measured spectrophotometrically at 545 nm. 2 ml of the RBC suspension was added to equal volume of saline as a negative control (0% lysis), while 1 % Triton X100 was used as a positive control (100% lysis). The hemolytic rates of the samples were calculated using the following equation [[7](#_ENREF_7)]:

$$Hemolytic rate=\frac{A_{t}-A_{nc}}{A_{pc}- A_{nc}} x 100 \% (3)$$

where A_t_ represents absorbance value of test sample, A_nc_ and A_pc_ stand for absorption value of negative and positive controls, respectively.

To mimic blood circulation conditions, the NPs were incubated with an equal volume of 10% FBS for 6 h at 37°C under mild stirring. At specified intervals, 50 µl of the mixture was taken then diluted in distilled water (1:50 v/v) to be re-evaluated for their PS and PDI.

1. ***In vitro* cytotoxicity**

The *in vitro* cytotoxicity of free RSV, free PMT, free RSV/PMT solution and different dual drug loaded QDs-BSA NPs against Human breast cancer MCF-7 and MDA-MB-231 cells. Human breast cancer MCF-7 and MDA-MB-231 cells were maintained in Dulbecco’s modified eagle medium (DMEM) containing 10% FBS in a CO_2_ incubator (5% CO_2_ at 37°C). Cells were seeded at a density of 5 × 10^3^/well in a 96-well plate containing 100 μl of DMEM enriched and allowed to adhere to the plate overnight. The cytotoxic activities of drugs either free or nano encapsulated against both cells was evaluated by the MTT assay. Therefore, the medium was replaced by fresh medium containing various concentrations of the drugs either as a solution or the prepared NPs and incubated for another 24 h. A stock solution of RSV, PMT and combination of both were prepared individually in DMSO using concentration of 1 mg/ml and stored at -70º C. Concentration of DMSO in the medium was kept <0.1%. The culture medium was then replaced with 100 μl of MTT solution (0.5 mg/ml in DMEM) then incubated for further 4 h at 37°C in the dark. After removal of MTT solution by aspiration, 100 μl of DMSO were added to the wells to dissolve MTT-formazan crystals formed after internalization of MTT by live cells and maintained in agitation for 15 min. Absorbance of the converted dye was measured at a wavelength of 570 nm with background subtraction at 690 nm using a microplate reader (Model 550, Bio-Rad, USA). The relative cell viability was expressed as a percentage of the untreated control wells. The inhibitory concentration (IC_50_) values were determined using Origin 8.0 (Origin Lab, Northampton, MA) according to the fitted data.

1. **Cellular-uptake study**

MCF-7 cells were seeded on glass cover slips placed in six-well culture plates overnight at a density of 1.8 × 10^5^ cells. The medium was then replaced with fresh medium adjusted to pH 7.4, which mixed with the prepared targeted and non-targeted QDs-BSA NPs and free QDs. After incubation, the cellular uptake was terminated by removing the medium and washing the cells three times with cold PBS. After that, the cells were fixed with 4% paraformaldehyde solution and then the cover slips were then mounted onto microscope slides using 2-(4-ethoxyphenyl)-6-[6-(4-methylpiperazin-1-yl)-1*H*-benzimidazol-2-yl]-1*H*-benzimidazole (Hoechst) containing (DPX) as mounting medium. Imaging analysis was performed via confocal laser scanning microscopy (LEICA, DMi8, Mannheim/Wetzlar, Germany) using the following filter set; Excitation wavelength: 450 nm, and emission wavelength 530 nm.

1. ***In vivo* studies**

**8.1. Animals**

*In vivo* study was performed on 70 female mice (7-8 weeks, 25 ± 5 g) housed in stainless steel mesh cages in 10 groups of seven mice each, under standard conditions of light illumination, relative humidity, and temperature, and they had free access to standard laboratory food and water throughout the study. All procedures were performed according to a protocol approved by the Animal Care and Use Committee of the Faculty of Pharmacy, Alexandria University.

**8.2. Tumor growth biomarkers**

Excised tumors were homogenized using cold PBS to make a final 40% tissue homogenate. Angiogenesis was determined by measurement of the level of the angiogenic factor; vascular endothelial growth factor (VEGF-1) using "VEGF-1 ELISA Kit" (RayBio Tech Inc., USA). Apoptosis induction was detected by measurement of tissue caspase-3 level using "Caspase-3 (Casp-3) ELISA Kit” (WKEA Med Supplies Corp., USA). The markers were quantified according to the manufacturer’s protocol.

**8.3. Histopathological analysis**

10% neutral formalin was used for fixation of the tumor samples for 24 h at room temperature. A 5 μm thick section were brought down to distilled water, stained with H & E for 5 min and 2 min, respectively, dehydrated in alcohol and mounted in Canada balsam, then examined microscopically. 10 random sections of each tumor were examined (×40) to determine the necrosis score on a scale from 1 to 4. The necrosis in the excised mammary tumor was assessed by examining 10 random sections (×40) from each excised tumor and scoring on a scale from 1 to 4 for the following criterion: Score 4; section in poorly differentiated tumor showing >50% necrosis, Score 3; section in poorly differentiated tumor showing about 35% necrosis, Score 2; section in poorly differentiated tumor showing about 25% necrosis, Score 1; section in poorly differentiated tumor showing about 10% necrosis. The mean value of all 10 scores was computed for each excised tumor and expressed as Mean of necrosis scale ± S.E.

**8.4. Immunohistochemical analysis**

Neutral buffered formalin fixed tissue was embedded in paraffin. Then, tissues section (5 mm thick) was prepared using a microtome and mounted on slides. The analysis was done within 24 h of the sections were being cut. Sections were deparaffinized in xylene, rehydrated in graded alcohols (100%, 95% and 75% v/v) and washed in distilled water, the endogenous peroxidase activity was quenched with 0.01% H_2_O_2_. Sections for Ki-67 were treated with 0.05% trypsin, 0.05% CaCl_2_ in Tris-HCl (pH 7.6) for 5 min at 37ºC. Antigen retrieval was done by microwaving the sections in 10mM/L citric acid (pH 6.0) for 30 min. The slides were washed thrice in PBS and blocked with 10% normal horse serum for 30 min. Tissue sections were then incubated with pre-diluted (1:50) mouse anti-Ki-67 monoclonal antibody (Santa Cruz Biotechnology Inc., cat. No. sc-23900) for 3 h at 4ºC. After being washed thrice with PBS, the sections were incubated with biotinylated goat anti-mouse IgG antibodies (1:500) (Abcam, USA, cat. No. ab-7067) for for 30 min at room temperature. The slides were then washed with PBS and labeled using avidin peroxidase conjugate (1:25) (Abcam, USA, cat. No. ab-59653) for 30 min at room temperature and then washed with PBS. Immunoreactivity was determined using diaminobenzidine (DAB) as the final chromogen. Finally, sections were counterstained with Meyer’s hematoxylin, dehydrated through a sequence of increasing concentrations of alcohol, cleared in xylene and mounted with epoxidic medium [[8](#_ENREF_8)].

**Quantification of the proliferative marker Ki-67 by image analysis technique**

Using digital image analysis technique, Ki-67 immunohistochemical detection signals were quantified. The technique involved the transformation of captured colored images into a grey scale by changing image type from colored type into bit-8 type. The area of immunohistochemical reaction to be measured was selected using oval selection tools. The obtained image was then analyzed by obtaining a histogram list of number of pixels of black and white areas. The degree of the reaction positivity was estimated by the percentage of black pixels in the binary image compared to the total number of pixels of selected area.

**8.5. Tissue localization of NPs**

The tumors of the mice were harvested, immediately washed twice with normal saline, weighed, fixed in 10% neutral formalin for 24 h at room temperature and sectioned at 5 μm. Tissues were treated with 0.25% Triton X-100 (in PBS) for 15 min and washed with PBS buffer. The cover slips were then mounted onto microscope slides and stained using 2-(4-ethoxyphenyl)-6-[6-(4-methylpiperazin-1-yl)-1*H*-benzimidazol-2-yl]-1*H*-benzimidazole (Hoechst) containing (DPX) as mounting medium. The distribution of BSA-QDs NPs was analyzed using the confocal laser scanning microscopy using the following filter set; Excitation wavelength: 450 nm, and emission wavelength 530 nm.

**8.6. Immunogenicity of the nano-delivery system**

Wells of high binding ELISA plates (Greiner Bio-one, Germany) were coated with 100 µl of the blank BSA NPs (10 µg/ml BSA w/v) overnight at 4°C. The wells were subsequently washed three times with PBS and blocked with blocking buffer (5% skimmed milk in PBS) for 3 h at room temperature. The wells were washed and diluted serum samples (1/25 dilution) from different treated and control animal groups were added to different wells for 1 h at 37°C with mild shaking. Thereafter, wells were washed thrice with PBS and 50 µl peroxidase-conjugated anti-mouse IgG antibody were added for 1 h. ELISA plate was subsequently washed and 100 µl of 3,3',5,5'-Tetramethylbenzidine (TMB) substrate were added for 10-15 min. Afterwards, 50 µl of 1 M sulfuric acid were added to stop the reaction. Absorbance was measured at 450 nm using ELISA plate reader (Biotek Inc., USA).

1. **Statistical analysis**

The *in vitro* and *in vivo* results were statistically analyzed using Student’s t-test (P<0.05) (GraphPad Software, CA, USA). On the other hand, the *in vivo* anti-tumor efficacy was analyzed via IBM SPSS Software package (version 20). Analysis of variance test (ANOVA) and Turkey’s Multiple Comparison test were utilized for pair-wise statistical comparisons between the investigated groups. Significance of the results was judged at the 5% level.

1. **Results and discussion**

**10.1. Characterization of RSV-PC Complex**

RSV-PC complex displayed an average size of 191.5±2.3 nm and zeta potential of -32.8±1.2 mV. The lyophilization yield was 82% w/w with drug content equal to 99% w/w. The FTIR spectrum of RSV showed a strong broad peak at the range of 3450-3100 cm^−1^ assigned to the three hydroxyl groups of RSV. The peaks at 1607–1445 cm^−1^ can be attributed to skeleton vibration of the benzene ring and conjugated olefin, and the peak at 965 cm_−1_ is a typical absorption peak of trans-olefin [[9](#_ENREF_9)].

**10.2. FTIR Spectroscopy**

The FTIR spectrum of PMT showed a characteristic broad band at the range of 3600-2600 cm-1 and a signal at 1703 cm-1 indicating the presence of COOH group [[10](#_ENREF_10)]. The IR peaks of pure BSA were reported at 3430, 3062, 1652, and 1531 cm^−1^ assigned to the stretching vibration of – OH, amide A (mainly –NH stretching vibration), amide I (mainly C=O stretching vibrations), and amide II (the coupling of bending vibrate of N–H and stretching vibrate of C–N) bands, respectively (Figure S4B).[[11](#_ENREF_11)]

**10.3. Body weight measurement**

Body weight loss of the experimental animals considered as an initial parameter to indicate the drug toxicity. So, body weights of each group were determined once per week along the experiment duration and shown as average of duplicate measurements (n=7 for each group). In the current study, in case of free PMT, RSV and free combined RSV/PMT-treated mice groups, the starting body weight average (BWA) was 27, 25.9 and 26 g, respectively; while at end of the study their weights have been reduced to 22.1, 20.6 and 20.7, respectively. The body weight loss observed in these free drug groups may be attributed to considerable toxicity along with reduced therapeutic efficacy of free drugs when compared with our prepared nano-formulations. On the other hand, insignificant body weight loss was observed in almost of other drug-loaded NPs-treated mice groups. At the beginning of the study, the drug-loaded NPs (F2, F3 and F4)- treated groups showed BWA of 23.4, 25.5 and 28 g, respectively while by the end of the treatment course, BWA was 22.5, 23.9 and 26.3 g, respectively indicating that our prepared nano-formulations succeeded to reduce the toxicity of free drugs throughout experimental period (Table S1).

**Table S1:** Body weight average (BWA) of each mice group throughout the study.

| Mice group | 1^st^ day | 7^th^ day | 14^th^ day | 21^st^ day |
| --- | --- | --- | --- | --- |
| Positive control | 23.8±1.2 | 24.7±2 | 24.95±1 | 25.3±1.6 |
| RSV | 25.9±1.4 | 24.3±0.9 | 23.4±1.9 | 20.6±1 |
| PMT | 27±1.7 | 26.1±1.1 | 24.98±2 | 22.1±2.2 |
| RSV/PMT | 26± 1.6 | 23.2±1.5 | 22±1.7 | 20.7±1.2 |
| (Blank NPs) | 27.2±2.1 | 26.8±1.8 | 27.9±1.5 | 28.5±1.3 |
| F2 (RSV/PMT-BSA-QDs NPs) | 23.46±1.1 | 23±1.4 | 22.2±1.6 | 22.5±1.2 |
| F3(Mann-targeted RSV/PMT BSA-QDs NPs) | 25.5±1.9 | 25±1.6 | 24.12±1.3 | 23.9±1.5 |
| F4(Mann-targeted RSV/PMT BSA NPs) | 28±2 | 27.5±1.6 | 26±2.1 | 26.3±1.9 |

**
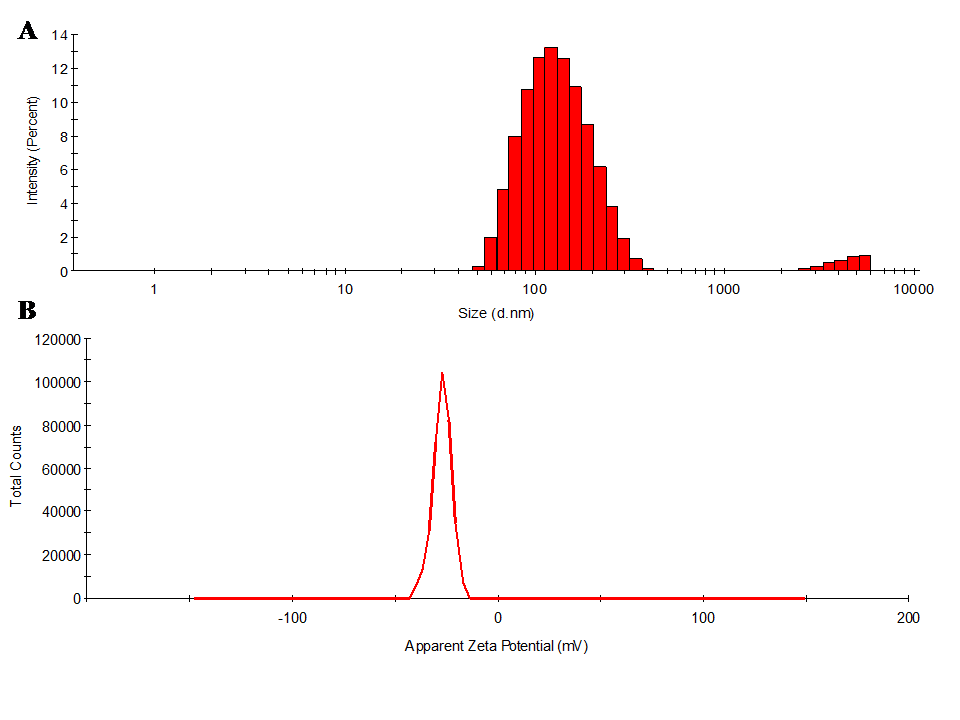
**

Figure S1: Physicochemical properties of the prepared RSV/PMT-BSA-QDs NPs (F2); size distribution diagram of non-targeted drug loaded BSA-QDs NPs (F2) (A) and their corresponding zeta potential distribution (B).

**
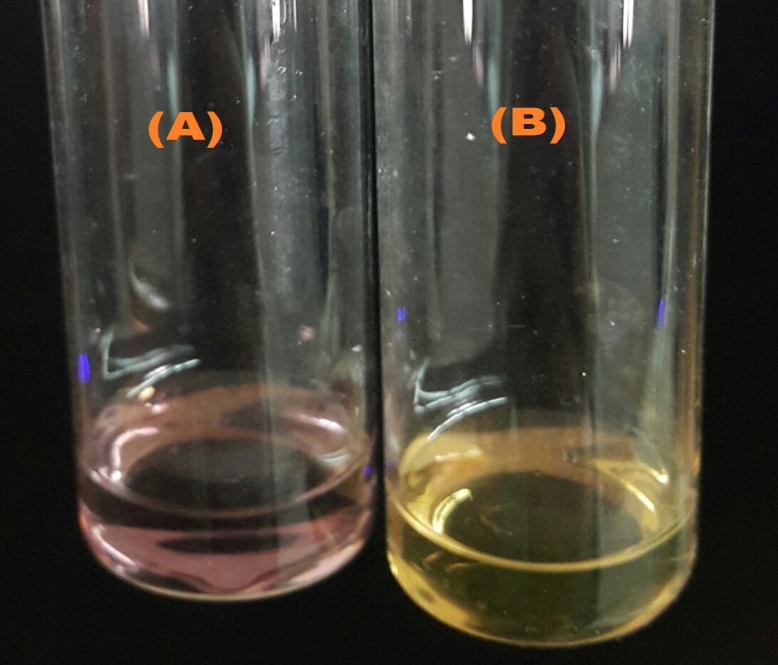
**

Figure S2: A photograph illustrating the phenol sulfuric acid test of BSA (A) and Mannose-BSA NPs conjugate (B).


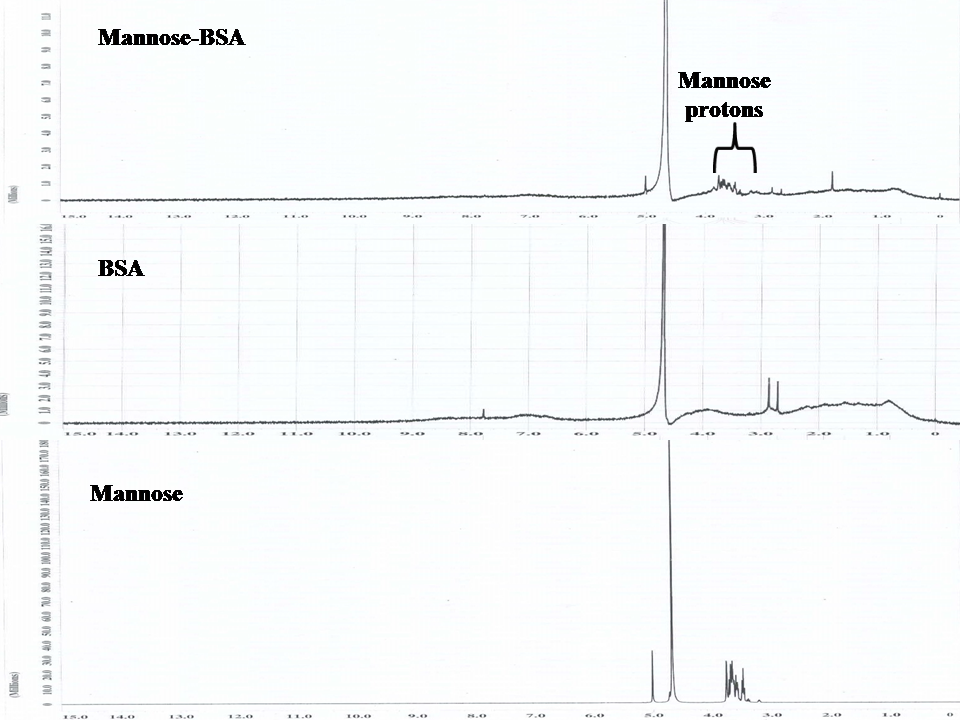


Figure S3: ^1^H-NMR spectra of mannose-BSA, BSA and mannose revealing the presence of mannose protons in the spectra of mannose-BSA conjugate.


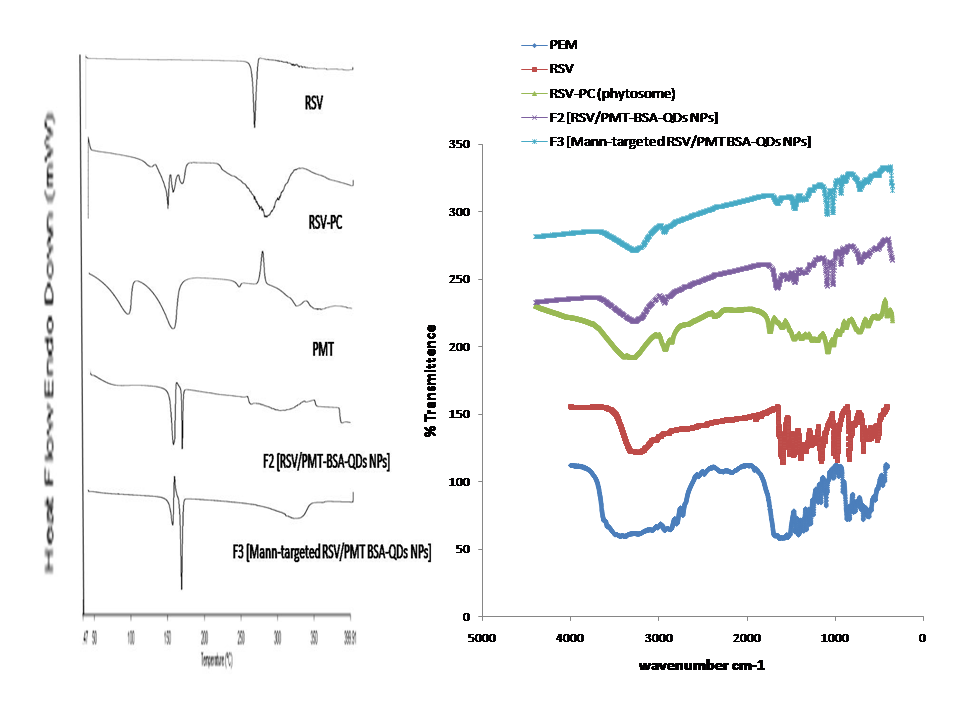


Figure S4: DSC thermogram of RSV, RSV-PC complex, PMT, RSV/PMT-BSA-QDs NPs (F2) and Mann-targeted RSV/PMT BSA-QDs NPs (F3) (A) and Fourier Transform Infrared (FTIR) spectra of RSV, RSV-PC complex, PMT, F2 and F3 (B).

**Figure S5***: In-vivo* anti-tumor efficacy showing change in body weights measurements of mice at indicated time-points along the experiment duration (C).

**References**

[1] N. Nakajima, Y. Ikada, Mechanism of amide formation by carbodiimide for bioconjugation in aqueous media, Bioconjugate chemistry, 6 (1995) 123-130.

[2] S.W. El-Far, M.W. Helmy, S.N. Khattab, A.A. Bekhit, A.A. Hussein, A.O. Elzoghby, Phytosomal bilayer-enveloped casein micelles for codelivery of monascus yellow pigments and resveratrol to breast cancer, Nanomedicine, 13 (2018) 481-499.

[3] N. Elgindy, K. Elkhodairy, A. Molokhia, A. Elzoghby, Biopolymeric microparticles combined with lyophilized monophase dispersions for controlled flutamide release, International journal of pharmaceutics, 411 (2011) 113-120.

[4] A.O. Elzoghby, B.Z. Vranic, W.M. Samy, N.A. Elgindy, Swellable floating tablet based on spray-dried casein nanoparticles: Near-infrared spectral characterization and floating matrix evaluation, International journal of pharmaceutics, 491 (2015) 113-122.

[5] A.O. Elzoghby, M.W. Helmy, W.M. Samy, N.A. Elgindy, Micellar delivery of flutamide via milk protein nanovehicles enhances its anti-tumor efficacy in androgen-dependent prostate cancer rat model, Pharmaceutical research, 30 (2013) 2654-2663.

[6] N. Elgindy, K. Elkhodairy, A. Molokhia, A. ElZoghby, Biopolymeric nanoparticles for oral protein delivery: design and in vitro evaluation, J Nanomed Nanotechnol, 2 (2011) 110.

[7] A.O. Elzoghby, S.K. Mostafa, M.W. Helmy, M.A. ElDemellawy, S.A. Sheweita, Superiority of aromatase inhibitor and cyclooxygenase-2 inhibitor combined delivery: hyaluronate-targeted versus PEGylated protamine nanocapsules for breast cancer therapy, International journal of pharmaceutics, 529 (2017) 178-192.

[8] A.O. Elzoghby, N.I. Saad, M.W. Helmy, W.M. Samy, N.A. Elgindy, Ionically-crosslinked milk protein nanoparticles as flutamide carriers for effective anticancer activity in prostate cancer-bearing rats, European Journal of Pharmaceutics and Biopharmaceutics, 85 (2013) 444-451.

[9] Y. Zhang, H. Song, Z. Shang, A. Chen, D. Huang, H. Zhao, H. Du, Amino acid-PEGylated resveratrol and its influence on solubility and the controlled release behavior, Biological and Pharmaceutical Bulletin, 37 (2014) 785-793.

[10] M. Vandana, S.K. Sahoo, Reduced folate carrier independent internalization of PEGylated pemetrexed: a potential nanomedicinal approach for breast cancer therapy, Molecular pharmaceutics, 9 (2012) 2828-2843.

[11] P. Huang, Z. Li, H. Hu, D. Cui, Synthesis and characterization of bovine serum albumin-conjugated copper sulfide nanocomposites, Journal of Nanomaterials, 2010 (2010) 33.
